# Midbrain circuit regulation of individual alcohol drinking behaviors in mice

**DOI:** 10.1038/s41467-017-02365-8

**Published:** 2017-12-20

**Authors:** Barbara Juarez, Carole Morel, Stacy M. Ku, Yutong Liu, Hongxing Zhang, Sarah Montgomery, Hilledna Gregoire, Efrain Ribeiro, Marshall Crumiller, Ciorana Roman-Ortiz, Jessica J. Walsh, Kelcy Jackson, Denise E. Croote, Yingbo Zhu, Song Zhang, Leandro F. Vendruscolo, Scott Edwards, Amanda Roberts, Georgia E. Hodes, Yongke Lu, Erin S. Calipari, Dipesh Chaudhury, Allyson K. Friedman, Ming-Hu Han

**Affiliations:** 1 Department of Pharmacological Sciences and Institute for Systems Biomedicine, Icahn School of Medicine at Mount Sinai, New York, NY 10029 USA; 2 Fishberg Department of Neuroscience and Friedman Brain Institute, Icahn School of Medicine at Mount Sinai, New York, NY 10029 USA; 3 Jiangsu Province Key Laboratory of Anesthesiology, Xuzhou Medical University, Xuzhou, 221002 Jiangsu China; 4 Intramural Research Program, National Institute on Drug Abuse, National Institutes of Health, Baltimore, MD 21224 USA; 5 Department of Physiology, Alcohol and Drug Abuse Center of Excellence, Neuroscience Center of Excellence, Louisiana State University Health Sciences Center, New Orleans, LA 70112 USA; 6 Department of Molecular and Cellular Neuroscience, The Scripps Research Institute, San Diego, CA 92037 USA; 7 Department of Medicine, Icahn School of Medicine at Mount Sinai, New York, CA 10029 USA; 8 Department of Health Sciences, College of Public Health, East Tennessee State University, Johnson City, TN 37614 USA; 9 Department of Pharmacology, Vanderbilt University, Nashville, TN 37232 USA; 10 Division of Science, New York University Abu Dhabi (NYUAD), Saadiyat Island Campus, Abu Dhabi, PO Box 129188 United Arab Emirates; 11 Department of Biological Sciences, Hunter College, City University of New York, New York, NY 10065 USA

## Abstract

Alcohol-use disorder (AUD) is the most prevalent substance-use disorder worldwide. There is substantial individual variability in alcohol drinking behaviors in the population, the neural circuit mechanisms of which remain elusive. Utilizing in vivo electrophysiological techniques, we find that low alcohol drinking (LAD) mice have dramatically higher ventral tegmental area (VTA) dopamine neuron firing and burst activity. Unexpectedly, VTA dopamine neuron activity in high alcohol drinking (HAD) mice does not differ from alcohol naive mice. Optogenetically enhancing VTA dopamine neuron burst activity in HAD mice decreases alcohol drinking behaviors. Circuit-specific recordings reveal that spontaneous activity of nucleus accumbens-projecting VTA (VTA-NAc) neurons is selectively higher in LAD mice. Specifically activating this projection is sufficient to reduce alcohol consumption in HAD mice. Furthermore, we uncover ionic and cellular mechanisms that suggest unique neuroadaptations between the alcohol drinking groups. Together, these data identify a neural circuit responsible for individual alcohol drinking behaviors.

## Abstract (web summary)

Mice exposed to a two-bottle alcohol choice paradigm can be divided into high and low drinking groups. Here, the authors show that stimulating VTA neurons to induce higher phasic activity patterns that are observed in low alcohol drinking mice, suppresses alcohol drinking in mice that are high alcohol drinking.

## Introduction

Alcohol-use disorder (AUD) is a debilitating addiction syndrome of ranging severity that causes tremendous personal and socioeconomic burdens^1,2^. Alcohol-use disorder is thought to be mediated by the repeated transition across three stages of escalated intake, withdrawal, and craving that are known to be regulated by specific neural circuits involved in drug reward, negative affect, and executive control^3^. Importantly, while a significant portion of individuals consume alcohol, only a subpopulation of these individuals consumes alcohol in a pathological manner to ultimately be diagnosed with an AUD. This is a phenomenon that is well-associated with alcohol, but the neural circuits that regulate individual alcohol consumption behaviors have yet to be elucidated^4^.

The mesocorticolimbic dopamine system consists of ventral tegmental area (VTA) dopamine neurons projecting to neural substrates involved in reward processing, including the nucleus accumbens (NAc) and medial prefrontal cortex (mPFC)^5,6^. VTA dopamine neurons display tonic, single spike activity or high frequency, burst/phasic activity, a firing pattern important in encoding behaviors associated with natural reward by increasing dopamine concentrations downstream^7,8^. Interestingly, these neurons show functional differences based on their downstream projection target^9,10^. Dysfunctions in neural activity of the dopaminergic circuit are known to be involved in the initial stages of drug addiction, including alcohol addiction^3,5,11^.

Acute ethanol (EtOH) activates VTA dopamine neurons to increase dopamine concentrations in downstream targets of the VTA circuit^12,13^. EtOH directly modulates VTA dopamine activity by acting on ion channels and receptors to increase dopamine firing activity and EtOH can also indirectly modulate VTA dopamine neurons by altering extrinsic inputs that contribute to VTA dopamine activity^14,15^. EtOH’s actions on VTA dopamine neurons and the role these neurons play in the initial rewarding and reinforcing properties of EtOH makes the VTA dopamine reward circuit an ideal system to investigate as a potential regulator of individual differences in alcohol drinking behaviors.

Understanding the neural mechanisms underlying individual drinking behaviors is of critical importance to the alcohol addiction field. Thus, to parse out individual differences in alcohol drinking behaviors and to probe the VTA dopaminergic reward circuit, we used a continuous access, two-bottle choice alcohol drinking paradigm that results in low and high alcohol drinking behaviors in isogenic C57BL/6J male mice. This paradigm involves the voluntary intake of alcohol, an individual behavior thought to be mediated by forebrain reward circuits, which are modulated by the mesocorticolimbic dopamine system^2,3^. Therefore, this paradigm provides one with an ideal model to determine how the VTA dopamine system regulates individual alcohol drinking behaviors. We performed in vivo recordings of putative VTA dopamine neurons between EtOH naive, low, and high alcohol drinking mice and discovered unique increases in in vivo dopaminergic firing in the low alcohol drinking population. Optogenetically increasing VTA dopamine neuron phasic/burst activity in high alcohol drinking mice significantly reduced individual alcohol drinking behaviors. Next, given the diversity of VTA dopamine neurons, we investigated how differential VTA dopamine neurons that project to the NAc or to the mPFC mediate individual alcohol drinking behaviors using circuit-dissecting electrophysiological and optogenetic approaches. Finally, we uncovered unique neurophysiological properties in the VTA-NAc circuit between low and high alcohol drinking mice that could underlie variable alcohol consumption behaviors.

## Results

### Mice can be parsed into low or high alcohol drinking groups

**Fig. 1:**
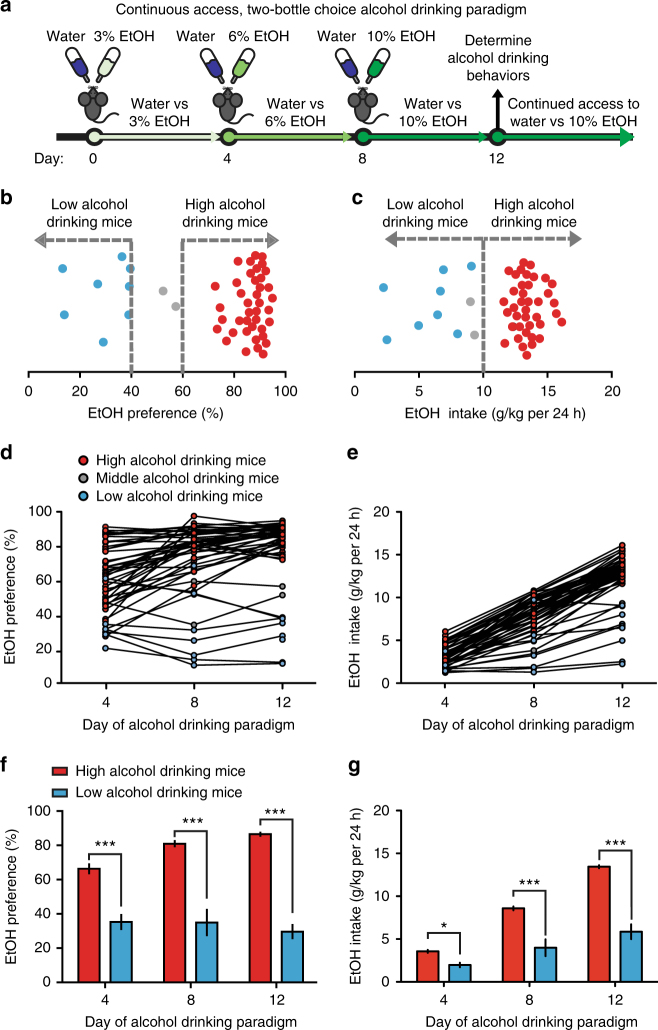
Individual alcohol drinking behaviors in isogenic C57BL/6J mice. **a** Individual alcohol drinking behaviors are determined following a continuous access, 12-day, 2-bottle choice alcohol drinking paradigm; mice then have continued access to water or 10% EtOH (v/v) until otherwise noted. **b** Representative distribution of EtOH preference on 12th day of alcohol drinking paradigm (*n* = 50). **c** Representative distribution of EtOH intake on 12th day of alcohol drinking paradigm (*n* = 50). **d** Individual EtOH preferences plotted across days of alcohol drinking paradigm. Data are EtOH preferences on 4th day of 3% EtOH (day 4), 4th day of 6% EtOH (day 8), and 4th day of 10% EtOH (day 12). **e** Individual EtOH intakes plotted across days of alcohol drinking paradigm. Data are 24 h EtOH intake behaviors on 4th day of 3% EtOH (day 4), 4th day of 6% EtOH (day 8), and 4th day of 10% EtOH (day 12). **f** Low and high alcohol drinking group EtOH preferences (two-way RM ANOVA: interaction effect *F*_(2,92)_ = 12.58, *P* < 0.001; drinking group effect *F*_(1,92)_ = 146.9, *P* < 0.001; Bonferroni post hoc test, ***P < 0.001. *n* = 8 mice; *n* = 40 mice). **g** Low and high alcohol drinking group EtOH intake over 24 h (two-way RM ANOVA: interaction effect *F*_(2,92)_ = 63.75, *P* < 0.001; drinking group effect *F*_(1,92)_ = 103.8, *P* < 0.001; Bonferroni post hoc test, *P < 0.05, ***P < 0.001. *n* = 8 mice; *n* = 40 mice). Data represented as mean + S.E.M.

Isogenic C57BL/10 mice have been reported to display individual alcohol preferences, however, the C57BL/6J population is commonly used for intrastrain neurophysiological and behavioral comparisons of individual responses to stress and drug abuse^16,17^. Thus, we utilized a continuous access (24 h), two-bottle choice alcohol drinking paradigm to generate individual low and high alcohol drinking C57BL/6J mice (Fig. 1a). Here, 8-week-old C57BL/6J male mice were allowed to voluntarily consume water and increasing concentrations of alcohol (4 days each concentration: 3%, 6%, 10% v/v EtOH) across 12 days. Alcohol drinking behaviors were determined on the fourth day of access to 10% EtOH and water (12th day). Experimental mice were then maintained on this two-bottle choice between water and 10% EtOH, until otherwise noted.

The majority (75–80% of the population) of C57BL/6J mice displayed preferences above 60% for the EtOH bottle, whereas ~10% of C57BL/6J mice displayed preferences of 40% or lower for the EtOH bottle on the 12th day of the alcohol drinking paradigm (Fig. 1b). The majority of the mice displayed high alcohol intake (consumption) behaviors (above 10 g EtOH/kg per 24 h), whereas a proportion of mice displayed low EtOH intake behaviors (<10 g EtOH/kg per 24 h, Fig. 1c). Thus, we classified low alcohol drinking mice as having both <40% EtOH preference and <10 g/kg per 24 h EtOH intake. High alcohol drinking mice were classified as having an EtOH preference of >60% and EtOH intake of >10 g EtOH/kg per 24 h.

Alcohol drinking behaviors from the fourth, eighth, and twelfth day of the alcohol drinking paradigm revealed that EtOH preference and intake behaviors had changed throughout the drinking paradigm and stabilized by the 12th day (Fig. 1d, e). Low alcohol drinking mice had EtOH preference and EtOH intake behaviors significantly less than high alcohol drinking mice (Fig. 1f, g). Additionally, total liquid intake and body weight was not significantly different between alcohol drinking groups (Supplementary Fig. 1a, b). EtOH preference remained consistent across the alcohol drinking paradigm with small populations in a cohort changing their preferences (Supplemental Fig. 2a). Moreover, the preference for a bitter solution was not significantly different between the alcohol drinking groups (Supplemental Fig. 2b). Consistent with drinking levels, low alcohol drinking mice had a significantly lower blood EtOH concentration (BEC) than high alcohol drinking mice (Supplementary Fig. 2c).

With this voluntary alcohol drinking paradigm, we have generated two alcohol drinking populations that display stable individual differences in EtOH preference and intake behaviors. To determine the neural mechanisms regulating these differences, we turned to in vivo electrophysiology of the dopaminergic system.

### Distinct VTA neuronal activity in alcohol drinking groups

**Fig. 2:**
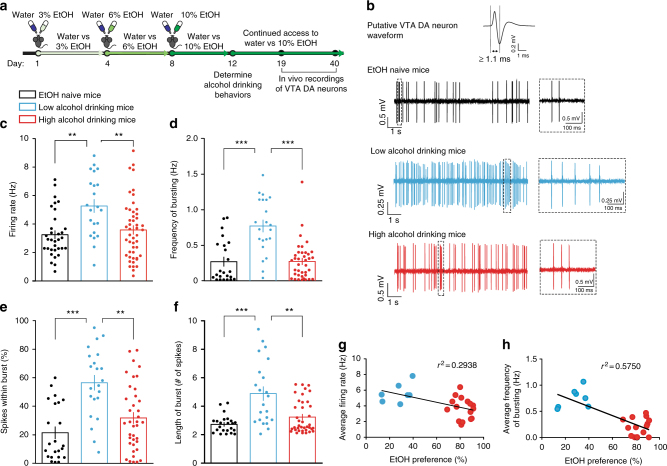
Low alcohol drinking mice have higher VTA dopamine neuron activity in vivo. **a** Timeline for experiments and schematic of single-unit recordings. **b** Representative in vivo VTA dopamine (DA) neuron firing traces for EtOH naive, low alcohol drinking, and high alcohol drinking mice, where dashed boxes show representative bursts. **c** VTA DA neuron firing rate in vivo (Kruskal–Wallis: *K*_(3)_ = 12.74, *P* < 0.01; post hoc Dunn’s multiple comparison test, **P < 0.01. *n* = 33 cells from 7 mice; *n* = 22 cells from 7 mice; *n* = 48 cells from 16 mice). **d** VTA DA neuron frequency of in vivo bursting (Kruskal–Wallis: *K*_(3)_ = 23.88, *P* < 0.0001; post hoc Dunn’s multiple comparison test, ***P < 0.001. *n* = 23 cells from 7 mice; *n* = 22 cells from 7 mice; *n* = 38 cells from 15 mice). **e** VTA DA neuron percentage of spikes within a burst in vivo (Kruskal–Wallis: *K*_(3)_ = 21.02, *P* < 0.0001; post hoc Dunn’s multiple comparison test, **P < 0.01, ***P < 0.001. *n* = 23 cells from 7 mice; *n* = 22 cells from 7 mice; *n* = 38 cells from 15 mice). **f** Average length of burst in VTA DA neurons in vivo (Kruskal–Wallis: *K*_(3)_ = 16.34, *P* < 0.001; post hoc Dunn’s multiple comparison test, ***P < 0.001, **P < 0.01. *n* = 23 cells from 7 mice; *n* = 22 cells from 7 mice; *n* = 38 cells from 15 mice). **g** Correlation between average VTA DA neuron in vivo firing rate and EtOH preference from individual alcohol drinking mice (*r*^2^ = 0.2938, *P* < 0.01. *n* = 23 mice). **h** Correlation between average VTA DA neuron in vivo frequency of bursting and EtOH preference from individual alcohol drinking mice (*r*^2^ = 0.5750, *P* < 0.01. *n* = 22 mice). Data represented as mean + S.E.M.

We examined whether there were differences in the in vivo firing properties of putative VTA dopamine neurons in EtOH naive, low alcohol drinking and high alcohol drinking mice exposed to a two-bottle choice alcohol drinking paradigm until the day of recording (Fig. 2a). We discovered that low alcohol drinking mice displayed significantly higher firing rates in putative VTA dopamine neurons when compared to EtOH naive and high alcohol drinking mice; high alcohol drinking mice, however, had firing rates similar to that of EtOH naive mice (Fig. 2b, c).

In vivo electrophysiology provides information not only on firing rate, but also on phasic/burst activity. This occurrence of fast, multispike activity in VTA dopamine neurons is known to be involved in the encoding of incentive salience properties of drugs of abuse^7,18,19^. Using standard electrophysiological criteria, we sought to determine the bursting activity of putative VTA dopamine neurons between the three groups^7,20,21^.

Putative VTA dopamine neurons of high alcohol drinking mice were not different in the frequency of bursting when compared to neurons of EtOH naive mice (Fig. 2b, d). This was complemented with no difference in the percentage of spikes within bursts (SWBs) (Fig. 2b, e). This suggests that a majority of putative VTA dopamine neuron activity in high alcohol drinking mice remains in an irregular, tonic state, similar to that of EtOH naive mice, even after the exposure to alcohol. However, low alcohol drinking mice had significantly higher frequency of bursting, percentage of SWB, and longer average length of bursts in putative VTA dopamine neurons when compared to EtOH naive mice and high alcohol drinking mice (Fig. 2b, d–f).

We further discovered that the average firing rate from each individual alcohol drinking mouse was negatively correlated with its own alcohol drinking behavior (Fig. 2g). We uncovered an even stronger correlation between average burst frequency and alcohol drinking behaviors in individual mice (Fig. 2h). These correlations led us to ask whether increased VTA dopaminergic neuron phasic/burst activity was causally linked to lower alcohol drinking behaviors and whether we could reduce alcohol consumption in high alcohol drinking mice by optogenetically stimulating VTA dopamine neurons.

### Activation of VTA dopamine neurons in high drinking mice

**Fig. 3:**
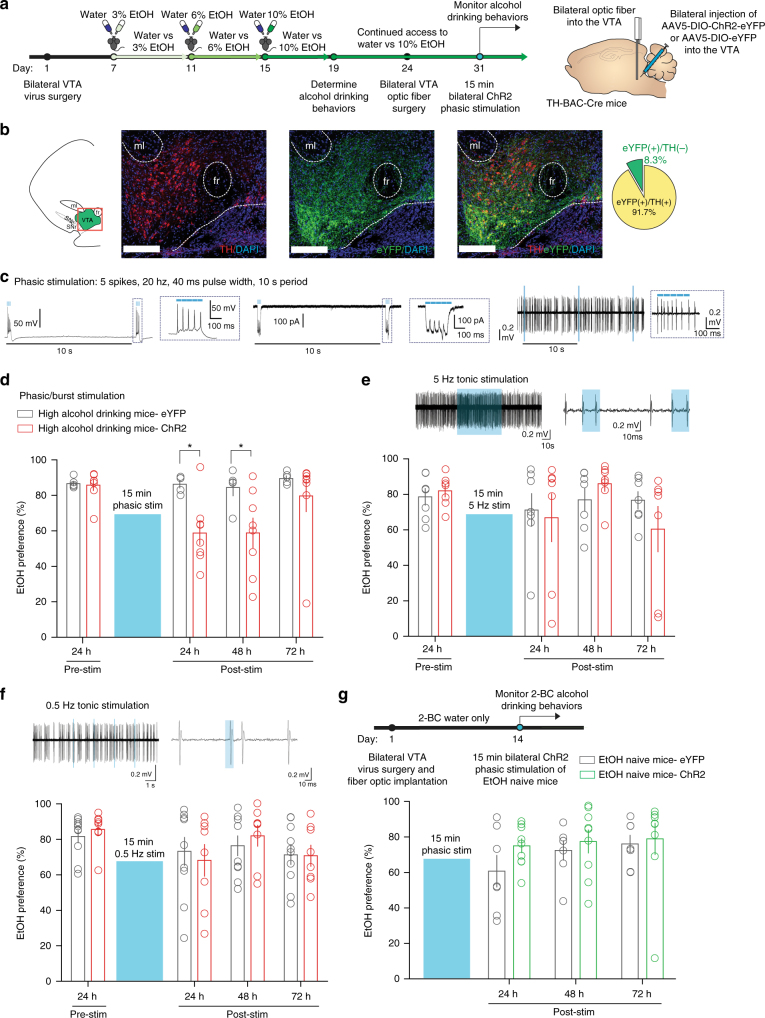
Phasically activating VTA dopamine neurons reduces high alcohol drinking behaviors. **a** Timeline of experiments and schematic of surgeries in *TH-BAC-Cre* mice. High alcohol drinking *TH-BAC-Cre* mice expressing either AAV5-DIO-eYFP (eYFP, control vector) or AAV5-DIO-ChR2-eYFP (ChR2) were selected following the alcohol drinking paradigm to undergo bilateral optic ferrule implantation into the VTA followed by phasic optical stimulation. **b** Colocalization of AAV-DIO-ChR2-eYFP in TH^+^ neurons of the VTA. *n* = 374 cells from 8 mice, scale bar = 150 μm. **c** Electrophysiological validation of phasic light stimulation via ChR2 in VTA dopamine (DA) neurons eliciting action potentials (left) and (middle) inward currents in vitro and burst firing in vivo. **d** EtOH preference of high alcohol drinking mice following 15 min phasic stimulation of VTA DA neurons (two-way RM ANOVA: interaction effect *F*_(3,33)_ = 2.922, *P* < 0.05; drinking group effect *F*_(1,33)_ = 5.765, *P* < 0.05; time effect *F*_(3,33)_ = 4.258, *P* < 0.05; Bonferroni post hoc test, eYFP vs ChR2. *P < 0.05 at 24 and 48 h. *n* = 5 mice; *n* = 8 mice). **e** (Top) In vivo validation of 5 Hz stimulation in VTA DA neurons and (bottom) EtOH preference between eYFP and ChR2 high alcohol drinking mice 24 h following 15 min 5 Hz stimulation of VTA DA neurons (two-way RM ANOVA: interaction effect *F*_(3,36)_ = 1.197, *P* > 0.05; drinking group effect *F*_(1,36)_ = 0.06571, *P* > 0.05; time effect *F*_(3,36)_ = 1.953, *P* > 0.05; Bonferroni post hoc test, *P* > 0.05. *n* = 7 mice; *n* = 7 mice). **f** (Top) In vivo validation of 0.5 Hz stimulation in VTA DA neurons and (bottom) EtOH preference between eYFP and ChR2 high alcohol drinking mice 24 h following 15 min 0.5 Hz stimulation of VTA DA neurons (two-way RM ANOVA: interaction effect *F*_(3,48)_ = 0.4922, *P* > 0.05; drinking group effect *F*_(1,48)_ = 0.02455, *P* > 0.05; time effect *F*_(3,48)_ = 3.451, *P* < 0.05; Bonferroni post hoc test, *P* > 0.05. *n* = 10 mice; *n* = 8 mice). **g** (Top) Timeline and schematic, *TH-BAC-Cre* mice received AAV5-DIO-eYFP (eYFP, control vector) or AAV5-DIO-ChR2-eYFP (ChR2), and bilateral optic ferrule implantation into the VTA. (Bottom) EtOH preference between eYFP and ChR2 EtOH naive mice 24 h following 15 min 5 Hz stimulation of VTA DA neurons (two-way RM ANOVA: interaction effect *F*_(2,28)_ = 0.7363, *P* > 0.05; drinking group effect *F*_(1,28)_ = 0.9406, *P* > 0.05; time effect *F*_(2,28)_ = 1.981, *P* > 0.05; Bonferroni post hoc test, *P* > 0.05. *n* = 7 mice; *n* = 9 mice). Data represented as mean + S.E.M.

Next, we used cell-type specific optogenetic manipulation of VTA dopamine neurons in high alcohol drinking mice to investigate whether increased phasic VTA dopaminergic activity contributes to low alcohol drinking behaviors. The tyrosine hydroxylase (TH, a rate limiting enzyme in dopamine synthesis)-BAC-Cre-recombinase (Cre) mouse line^22^, which has been backcrossed to the C57BL/6J line, has been used for specific targeting of Cre-inducible genes to TH^+^ populations^23–25^. Here, we injected Cre-inducible, blue-light (473 nm) activated, excitatory channelrhodopsin-2 (ChR2) with an enhanced yellow fluorescent protein (eYFP) or a Cre-inducible eYFP only (control) expressed in an AAV5 viral vector (AAV5-EF1α-DIO-hChR2-eYFP or AAV5-EF1α-DIO-eYFP, University of North Carolina) into the VTA of *TH-BAC-Cre* mice. Our own immunohistochemical investigation confirmed colocalization of Cre-inducible eYFP to TH^+^ populations (Fig. 3a, b; 374 cells from 8 mice), similar to previous reports^22,23,25^.

Next, using 8-week-old *TH-BAC-Cre* mice, we first determined and established individual alcohol drinking behaviors following the alcohol drinking paradigm in these transgenic mice (Supplementary Fig. 3a–f). *TH-BAC-Cre* mice also develop individual alcohol drinking behaviors by the 12th day: significant differences in EtOH preference between the two groups occurred throughout the measured three time points, whereas EtOH intake was statistically different between the two groups on the 8th day and later. After this confirmation of individual alcohol drinking behaviors in *TH-BAC-Cre* mice, we bilaterally injected 7-week-old EtOH naive *TH-BAC-Cre* males with AAV5-EF1α-DIO-hChR2-eYFP or AAV5-EF1α-DIO-eYFP into the VTA (Fig. 3a). In a subset of mice, we validated that a phasic optical stimulation protocol (phasic stimulation: 5 spikes, 20 Hz, 40 ms pulse width, 10 ms spike interval, every 10 s), induces burst-like firing in vivo, as previously demonstrated^27^. This protocol was found to elicit action potentials and inward photocurrents in vitro and burst firing in vivo in VTA dopamine neurons of *TH-BAC-Cre* mice infected with AAV5-DIO-ChR2-eYFP (Fig. 3c).

Following 1 week of recovery from surgery for bilateral virus injection, 8-week-old *TH-BAC-Cre* mice began the 12-day alcohol drinking paradigm. High alcohol drinking mice expressing either ChR2 or eYFP were then selected to receive optic fiber ferrules bilaterally implanted above the VTA. High alcohol drinking behaviors in both eYFP and ChR2 groups were re-established following 1 week of surgical recovery and pre-stimulation alcohol drinking behaviors 24 h before optogenetic manipulations were used for comparison (pre-stim, Fig. 3d). It is known that brief phasic stimulation of VTA dopamine neurons induces sustained molecular, electrophysiological, and behavioral alterations^23,27,45^. Therefore, high alcohol drinking-ChR2 and -eYFP *TH-BAC-Cre* mice received 15 min of bilateral phasic stimulation of VTA dopamine neurons in their home cage and then alcohol drinking behaviors were monitored.

Following a single 15 min phasic stimulation of VTA dopamine neurons of *TH-BAC-Cre* mice, EtOH preference and intake of high alcohol drinking ChR2 mice was significantly lower than that of high alcohol drinking eYFP mice 3 h following stimulation with a transient decrease in total liquid intake (Supplementary Fig. 4a–c). Interestingly, high alcohol drinking ChR2 mice maintained lower EtOH preference than high alcohol drinking eYFP mice 24 h following the 15 min phasic stimulation of VTA dopamine neurons (Fig. 3d, Supplementary Fig. 5a). Additionally, high alcohol drinking-ChR2 mice maintained lower EtOH preference 48 h following stimulation (Fig. 3d), with a restoration of high EtOH preference in the ChR2 group occurring 72 h following phasic activation of VTA dopamine neurons (Fig. 3d). In contrast to a transient decrease in 3 h total liquid intake, there were no differences between groups in total liquid intake between the ChR2 and the eYFP groups throughout the 24, 48, and 72 h time point measurements (Supplementary Fig. 6a).

In order to determine if the reduction in alcohol drinking behaviors was due to increased pattern-specific activity from burst firing induction or if it can be due to increased dopaminergic activity in general, we next stimulated a separate cohort of high alcohol drinking *TH-BAC-Cre* mice with ChR2 or eYFP in the VTA using a non-bursting, 5 Hz tonic stimulation protocol (5 Hz, 4 ms pulse width, 196 ms inter-spike interval) for 15 min (Fig. 3e, top), which has been previously used to alter alcohol drinking behaviors in rats during a limited access alcohol drinking session^26^, and then monitored alcohol drinking behaviors. We found no group differences in EtOH preference between eYFP and ChR2 high alcohol drinking mice at 3, 24, 48, and 72 h following 5 Hz tonic stimulation, similar to previous reports monitoring alcohol drinking after stimulation^26^ (Fig. 3e, Supplementary Fig. 4d–f). Additionally, we found no difference between groups on EtOH intake and total liquid intake. We did identify a significant effect of time on EtOH intake and total liquid intake in both eYFP and ChR2 groups (Supplementary Figs. 5b, 6b). Thus, these data suggest that the long-lasting reduction in alcohol drinking behaviors between groups is due to phasic-specific increases in VTA dopamine neuron firing.

Next, to control for the induction of action potentials in a 10 s period for 15 min from our phasic stimulation protocol, we performed another control optical stimulation paradigm in a separate cohort of *TH-BAC-Cre* high alcohol drinking mice where we optically stimulated VTA dopamine neurons in high alcohol drinking mice at a slower 0.5 Hz tonic stimulation for 15 min (Fig. 3f, 5 spikes, 0.5 Hz, 15 ms pulse width, 1.985 s inter-spike interval, every 10 s). We found no differences in EtOH preference and intake between the eYFP and ChR2 high alcohol drinking groups following this stimulation (Fig. 3f, Supplementary Figs. 4g, h, 5c). There were also no differences between groups in total liquid intake (Supplementary Figs. 4i, 6c).

Finally, in order to determine if the reduction in alcohol drinking behaviors observed with phasic stimulation of VTA dopamine neurons is dependent on alcohol experience, we performed optical stimulation in EtOH naive mice (Fig. 3g). We found no differences in EtOH preference or intake in EtOH naive between eYFP and ChR2 groups that received phasic stimulation of VTA DA neurons (Fig. 3g, Supplementary Figs. 4j, k, 5d) and no differences in total liquid intake between groups (Supplementary Figs. 4l, 6d).

Together, we discovered that phasically stimulating the VTA dopaminergic system of high alcohol drinking mice reduces alcohol drinking behaviors. However, recent research has demonstrated that VTA dopamine neurons have diverse functional properties based on their projection target in the mesocorticolimbic reward circuit^9,23,27,28^. Determining how these subpopulations of VTA dopamine neurons respond to drugs of abuse is now a critical avenue of therapeutic exploration^24^.

### Circuit-specific firing divergence in alcohol drinking mice

Ventral tegmental area dopamine neurons play a crucial role in tuning the properties of drugs of abuse in downstream substrates involved in reward and executive control. Pathological adaptations in VTA dopamine neurons firing after drug exposure have been shown to be mediators in drug addiction^3^. Therefore, because of dopamine’s role in the NAc and mPFC after drug exposure and ethanol’s ability to directly modulate VTA dopamine activity, we sought to determine how VTA neurons that project to the NAc and mPFC are altered after the alcohol drinking paradigm.

**Fig. 4:**
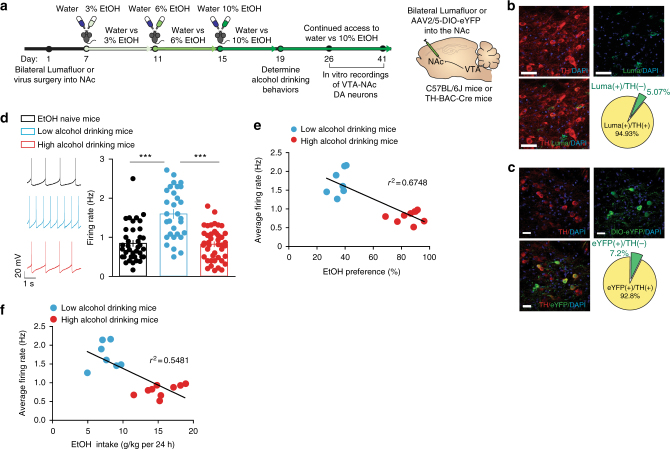
Alcohol drinking mice display different firing properties in the VTA-NAc pathway. **a** Timeline of circuit dissecting electrophysiological experiments. **b** Colocalization of retrograded microbeads (Lumafluor/Luma) from NAc in TH^+^ neurons of the VTA (*n* = 138 cells from 4 mice, scale bar = 50 μm). **c** Colocalization of retrograded AAV5-DIO-eYFP from NAc in TH^+^ (dopamine, DA) neurons of the VTA (*n* = 153 cells from 5 mice, scale bar = 25 μm). **d** Representative spontaneous firing rate traces (left) and grouped average firing rate (right) of VTA-NAc dopamine neurons in EtOH naive, low alcohol drinking, and high alcohol drinking mice (one-way ANOVA: *F*_(2, 12.87)_ = 25.74, *P* < 0.0001; post hoc Bonferroni-multiple comparison test, ***P < 0.001. *n* = 39 cells from 7 mice; *n* = 30 cells from 7 mice; *n* = 43 cells from 9 mice). **e** Correlation of average VTA-NAc neuron firing rate and EtOH preference from alcohol drinking mice (*r*^2^ = 0.6748, *P* < 0.0001. *n* = 16 mice). **f** Correlation between average VTA-NAc neuron firing rate and EtOH intake across 24 h from alcohol drinking mice (*r*^2^ = 0.5481, *P* < 0.001. *n* = 16 mice). Data represented as mean + S.E.M.

We performed whole-cell electrophysiological recordings in brain slices of the VTA from EtOH naive, low alcohol drinking, and high alcohol drinking mice to measure pathway-specific spontaneous firing activity. To isolate the VTA-NAc or VTA-mPFC pathways for these electrophysiological experiments, we used either retrograding microbeads (Lumafluors) injected into the NAc of C57BL/6J mice or retrograding Cre-inducible-eYFP viral vectors (AAV2/5-DIO-eYFP, University of Pennsylvania) injected into the NAc or mPFC of *TH-BAC-Cre* mice (Fig. 4a, Supplementary Fig. 7a). We found that NAc retrograded Lumafluor was ~95% colocalized to TH(+) neurons in the VTA (Fig. 4b, 138 cells from 4 mice) and NAc retrograded AAV2/5-DIO-eYFP was 92.8% colocalized with TH(+) neurons in the VTA (Fig. 4c, 153 cells from 5 mice). Baseline electrophysiological data from both approaches were not significantly different (Supplemental Fig. 8a). Thus, data from both approaches for VTA-NAc investigations were pooled.

We discovered that, similar to our in vivo data, the spontaneous firing rate of VTA-NAc neurons in low alcohol drinking mice was significantly higher when compared to EtOH naive mice and high alcohol drinking mice (Fig. 4d, Supplementary Fig. 8b). In contrast, VTA-mPFC dopamine neurons between both low and high alcohol drinking mice had no differences in activity when compared to EtOH naive mice (Supplemental Fig. 7b).

Furthermore, we found a significant negative correlation between average firing rate of VTA-NAc neurons and EtOH preference and EtOH intake (Fig. 4e, f). Together, these data suggest the VTA-NAc pathway specifically is a critical mediator of individual alcohol drinking behaviors. Accordingly, we sought to determine whether mimicking the increased activity we observed specifically in the VTA-NAc pathway of low alcohol drinking mice is sufficient to reduce high alcohol drinking behaviors in high alcohol drinking mice.

### Activating VTA-NAc neurons reduces alcohol drinking behaviors

**Fig. 5:**
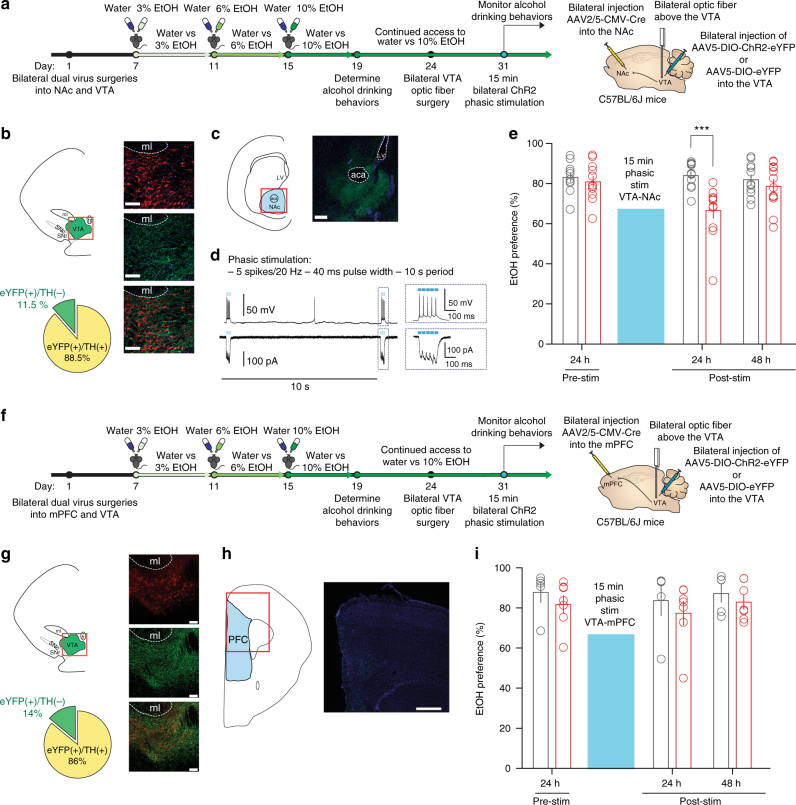
Phasically activating VTA-NAc neurons reduces high alcohol drinking behaviors. **a** Timeline of experiments and schematic of surgeries for VTA-NAc experiments. High alcohol drinking C57BL6J mice expressing either AAV5-DIO-eYFP (eYFP, control vector) or AAV5-DIO-ChR2-eYFP (ChR2) in VTA-NAc neurons were selected following the alcohol drinking paradigm to undergo bilateral optic ferrule implantation into the VTA followed by phasic optical stimulation. **b** VTA colocalization of AAV5-DIO-ChR2-EYFP expressed in VTA neurons projecting to the NAc with TH (*n* = 469 cells from 8 mice, scale bar = 100 μm). **c** Terminals in the NAc of VTA neurons containing AAV-DIO-ChR2-eYFP terminals from dual viral injections (scale bar = 250 μm). **d** Electrophysiological validation of phasic (burst) optical light stimulation via ChR2 in VTA-NAc neurons eliciting (top) action potentials and (bottom) inward currents. **e** EtOH preference of high alcohol drinking eYFP and ChR2 mice following 15 min of phasic VTA-NAc stimulation (two-way RM ANOVA: interaction effect *F*_(2,42)_ = 9.822, *P* < 0.001; drinking group effect *F*_(1,42)_ = 5.292, *P* < 0.05; time effect *F*_(2,42)_ = 6.518, *P* < 0.01; Bonferroni post hoc test, eYFP vs ChR2 at 24 h ***P < 0.001. *n* = 11 mice; *n* = 12 mice). **f** Timeline of experiments and schematic of surgeries for VTA-mPFC experiments. High alcohol drinking C57BL6J mice expressing either AAV5-DIO-eYFP (eYFP, control vector) or AAV5-DIO-ChR2-eYFP (ChR2) in VTA-mPFC neurons were selected following the alcohol drinking paradigm to undergo bilateral optic ferrule implantation into the VTA followed by phasic optical stimulation. **g** Colocalization of TH and AAV5-DIO-ChR2-EYFP expressed in VTA neurons projecting to the mPFC in the VTA (*n* = 300 cells from 3 mice, scale bar 100 μm). **h** Fibers from the VTA AAV-DIO-ChR2-eYFP in the mPFC (scale bar 500 μm). **i** EtOH preference behaviors of eYFP and ChR2 high alcohol drinking mice following phasic stimulation of the VTA-mPFC pathway (two-way RM ANOVA: interaction effect *F*_(2,20)_ = 0.05370, *P* > 0.05; drinking group effect *F*_(1,20)_ = 0.7967, *P* > 0.05; time effect *F*_(2,20)_ = 1.087, *P* > 0.0f; Bonferroni post hoc test, *P* > 0.05. *n* = 5 mice; *n* = 7 mice). Data represented as mean + S.E.M.

To determine whether specifically increasing the activity of the VTA-NAc pathway can reduce alcohol drinking behaviors, we first performed dual, bilateral viral injections of retrograding AAV2/5-CMV-Cre (University of Pennsylvania) into the NAc and AAV5-DIO-ChR2-eYFP or AAV-DIO-eYFP, as a control, into the VTA of C57BL/6J mice (Fig. 5a). We immunohistochemically confirmed expression of Cre-inducible ChR2-eYFP in cell bodies of the VTA (Fig. 5b, *n* = 469 cells from 8 mice) and in terminals in the NAc (Fig. 5c) 3 weeks following dual virus injections. We confirmed optical phasic stimulation parameters via patch-clamp electrophysiology (Fig. 5d). Following the alcohol drinking paradigm, high alcohol drinking C57BL/6J mice with either AAV5-DIO-ChR2-eYFP or AAV5-DIO-eYFP expressed in VTA-NAc neurons received bilateral optic fiber ferrule implantations above the VTA (Fig. 5a). High alcohol drinking behaviors were re-established in both eYFP and ChR2 mice 1 week following implantation (Fig. 5e, pre-stim).

We discovered that 15 min of phasically activating the VTA-NAc pathway bilaterally in high alcohol drinking C57BL/6J mice significantly reduces EtOH preference behavior in the high alcohol drinking-ChR2 group 3 h after phasic stimulation (Supplementary Fig. 9a) and 24 h after phasic stimulation (Fig. 5e), when compared to the eYFP group. However, there was no significant reduction in EtOH intake between groups 24 h after VTA-NAc stimulation (Supplementary Fig. 10a). Notably, VTA stimulation had no effects on total liquid consumed between groups (Supplementary Figs. 9c and 11a).

Interestingly, while we saw robust decreases in alcohol drinking behaviors at 3  and 24 h following phasic stimulation of the VTA-NAc pathway, high alcohol drinking behaviors of the ChR2 mice were restored 48 h following this stimulation (Fig. 5e). This suggests that while isolating phasic stimulation to the VTA-NAc pathway can significantly reduce alcohol drinking behaviors in high alcohol drinking mice, the effect is not as long-lasting as a cell-specific whole VTA approach as done with *TH-BAC-Cre* mice (Fig. 3d).

To determine if this pathway-specific reduction was a projection-specific effect, we also performed dual viral injections in EtOH naive mice to target the VTA-mPFC pathway (Fig. 5f). Mice then underwent the alcohol drinking paradigm and high alcohol drinking mice were selected to receive bilateral optical fibers implanted above the VTA (Fig. 5f). We confirmed colocalization of eYFP to TH^(+)^ cells and fibers in the mPFC (Fig. 5g, h; 300 cells from 3 mice). We found no group differences in EtOH preference throughout this paradigm (Fig. 5i). Moreover, no differences were found in EtOH intake in both groups (Supplemental Figs. 10b and 11b).

The revelation that the neurophysiological activity observed in VTA-NAc neurons is causal for eliciting specific alcohol drinking behaviors is striking. Therefore, we wanted to determine what intrinsic properties could underlie the neural activity between alcohol drinking groups.

### Intrinsic physiological properties of alcohol drinking mice

The discovery of unchanged firing of VTA dopamine neurons in high alcohol drinking mice following the alcohol drinking paradigm compared with naive mice both in vivo and in VTA-NAc neurons was unexpected, especially considering EtOH’s ability to activate VTA dopamine neurons directly^29,30^. Importantly, VTA dopamine neurons have the ability to maintain homeostasis and induce activity through actions on intrinsic ion channels^23^. Thus, we wanted to identify what were the possible ion channel alterations, intrinsic excitability changes, and consequences of EtOH responsivity underlying the firing properties we observed in VTA-NAc dopamine neurons. Of the varied ion channel targets on VTA dopamine neurons, hyperpolarization-activated cyclic nucleotide-gated channels that mediate excitatory hyperpolarization-activated currents (*I*_h_) are critical to regulating VTA dopamine neuron firing^23,31^ and *I*_h_ has been shown be modulated by repeated injections of EtOH^32,33^. Therefore, we determined the properties of excitatory *I*_h_ between the alcohol drinking groups for understanding how intrinsic properties on VTA dopamine neurons may mediate neurophysiological properties that underlie individual alcohol drinking behaviors.

**Fig. 6:**
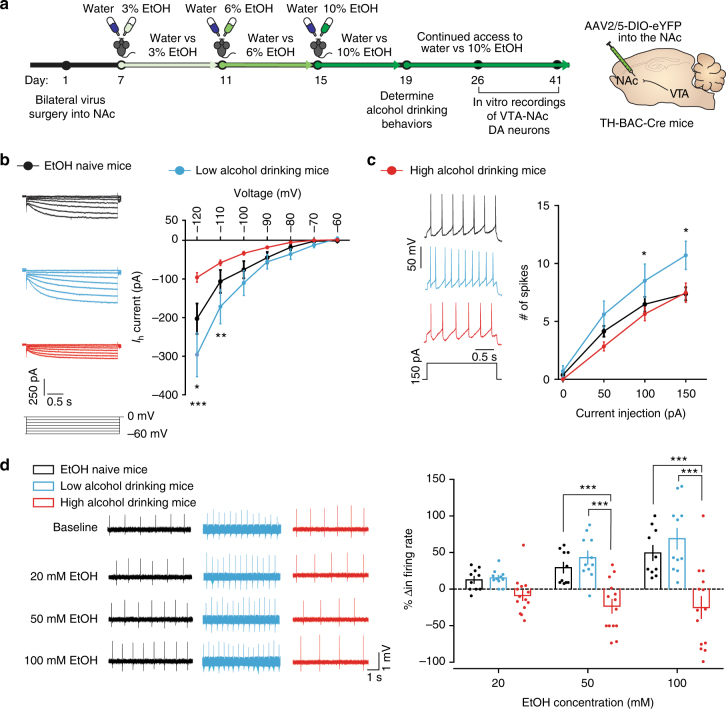
VTA-NAc neurons of alcohol drinking mice have different intrinsic properties and distinct responses to EtOH. **a** Timeline of experiments and schematic of surgery. **b** Representative *I*_h_ traces (left) and I–V graph of *I*_h_ (right). (two-way RM ANOVA: drinking group effect *F*_(2, 306)_ = 4.442, *P* < 0.05; interaction effect *F*_(12, 306)_ = 5.421, *P* < 0.0001. Bonferroni post hoc test *P < 0.05; **P < 0.01; ***P < 0.001. *n* = 23 cells from 7 mice; *n* = 10 cells from 5 mice; *n* = 21 cells from 6 mice). **c** Representative spike traces after current injection (left, 150 pA) and excitability data of VTA-NAc neurons from EtOH naive, low alcohol drinking, and high alcohol drinking mice (two-way RM ANOVA: drinking group effect *F*_(2, 159)_ = 4.551, *P* < 0.05; interaction effect *F*_(6, 159)_ = 1.839, *P* = 0.0948. Bonferroni post hoc test *P < 0.05. *n* = 21 cells from 5 mice; *n* = 10 cells from 3 mice; *n* = 25 cells from 5 mice). Data represented as mean + S.E.M. **d** Representative spontaneous firing rate traces during different bath applications of EtOH (left) and % change (Δ) in firing rate from baseline (responsivity) during bath application of 20, 50, and 100 mM EtOH in aCSF (right) (two-way RM ANOVA: drinking group effect *F*_(2, 64)_ = 15.18, *P* < 0.0001; EtOH concentration effect *F*_(2, 64)_ = 12.67, *P* < 0.0001; interaction effect *F*_(4, 64)_ = 10.30, *P* < 0.0001. Bonferroni post hoc test ***P < 0.001. *n* = 10 cells from 6 mice; *n* = 11 cells from 3 mice; *n* = 14 cells from 7 mice). Data represented as mean + S.E.M.

Using the circuit-dissecting techniques described above to isolate the VTA-NAc pathway for electrophysiological recordings, we recorded *I*_h_ in our experimental groups following the alcohol drinking paradigm (Fig. 6a). High firing VTA-NAc neurons of low alcohol drinking mice had significantly increased excitatory *I*_h_ following the alcohol drinking paradigm when compared to EtOH naive mice (Fig. 6b). In contrast, the VTA-NAc neurons in high alcohol drinking mice had significantly lower *I*_h_ than both low alcohol drinking and EtOH naive mice (Fig. 6b). Here, we identify a novel divergent characteristic of *I*_h_ that is associated with individual alcohol drinking behaviors.

With these ion channel observations, we furthered our investigation into probing intrinsic excitability between the three groups. We found that high alcohol drinking mice had intrinsic excitability comparable to EtOH naive mice, yet low alcohol drinking mice had significantly more action potentials elicited to current injections than the two other groups (Fig. 6c). This suggests the intrinsic properties of VTA-NAc neurons of low alcohol drinking mice are more excitable, however, VTA-NAc neurons of high alcohol drinking mice maintain EtOH naive-like excitable states even after the alcohol drinking paradigm.

### Altered ethanol responsivity in VTA-NAc neurons

Our ion channel data suggest that the VTA-NAc neurons of high alcohol drinking mice have undergone intrinsic neuroadaptations that maintain homeostatic stable firing and excitability while consuming high amounts of EtOH, a possible blunting of the dopaminergic system to the excitatory effects of EtOH. Thus, we sought to understand how the observed intrinsic neuronal adaptations observed in alcohol drinking mice affected the ability of these neurons to respond to the excitatory properties of EtOH. With the circuit dissecting techniques for in vitro electrophysiological studies following the alcohol drinking paradigm (Fig. 6a), we performed recordings to measure firing rate of VTA-NAc dopamine neurons during bath application of EtOH in artificial cerebrospinal fluid (aCSF), similar to previously published reports of EtOH responsivity in VTA dopamine neurons^34^. Baseline spontaneous activity (0 mM EtOH) of VTA-NAc dopamine neurons was significantly higher in low alcohol drinking mice. However, high alcohol drinking mice had baseline activity similar to EtOH naive mice, reflecting what we observed using in vivo and whole-cell in vitro electrophysiological methods (Supplementary Fig. 12a).

During bath application of EtOH, we discovered that the firing rate of VTA-NAc dopamine neurons of EtOH naive mice underwent dose-dependent, significant increases from baseline (% change in firing rate) (Fig. 6d, Supplemental Fig. 12b). VTA-NAc dopamine neurons of low alcohol drinking mice responded similarly to the excitatory effects of EtOH, undergoing significant increases in spontaneous activity from baseline (Fig. 6d, Supplemental Fig. 12b). However, we found that VTA-NAc dopamine neurons of high alcohol drinking mice failed to mount a response to the excitatory properties of EtOH (Fig. 6d, Supplemental Fig. 12b). These data further suggest that high alcohol drinking mice have undergone specific neuronal adaptations to maintain EtOH naive-like firing states, which blunts EtOH-induced effects in the brain’s reward circuit.

## Discussion

Here, we identified a key regulatory neural circuit and related mechanisms that mediate individual differences in alcohol drinking behaviors. We found that the low alcohol drinking subpopulation displayed higher activity of VTA dopamine neurons, specifically VTA-NAc neurons, whereas high alcohol drinking mice had activity similar to that of EtOH naive mice. Optical stimulation of these neurons, to induce higher phasic activity patterns observed in low alcohol drinking mice, suppressed alcohol drinking in the high alcohol drinking population. This higher activity was found to be specific to protocols using burst stimulation, a pattern of VTA dopamine firing known to release higher concentrations of dopamine downstream^8^. We discovered that *I*_h_, an excitatory current on VTA dopamine neurons responsible for spontaneous activity, may be a possible ion channel mechanism underlying these differences between alcohol drinking. The increased *I*_h_ we observed in VTA-NAc neurons of low alcohol drinking mice could contribute to their increased firing. In contrast, the significantly reduced *I*_h_ in VTA-NAc neurons of high alcohol drinking mice could be an adaptive response to the excitatory properties of EtOH to re-establish EtOH naive-like firing. Indeed, we discovered that high alcohol drinking mice failed to respond to the excitatory properties of EtOH, but low alcohol drinking mice maintained this ability. The blunted ability of EtOH to engage the VTA-NAc dopaminergic circuit in high alcohol drinking mice could drive these mice to consume more EtOH, which could contribute to the development of EtOH tolerance and AUD.

Alterations in dopaminergic signaling play a key role in addiction. Acute administration of EtOH initially increases VTA dopamine firing and dopamine release in the NAc^14^. However, EtOH withdrawal has been shown to reduce VTA dopamine firing^35,36^. Moreover, following chronic exposure to ethanol, reductions in dopamine release in the NAc were discovered^35,37^. These two components may contribute to impairments of dopamine signaling in addicted subjects. This dopamine deficit hypothesis is thought to be a key component for the transition into an addicted state^2,38^. Re-establishing dopamine signaling in the reward system may be a potential avenue for treatments to alcohol addiction. Indeed, overexpressing dopamine receptor 2 (D2R) in the NAc of rats lowered EtOH self-administration behaviors^39^. Moreover, in humans, non-addicted members from alcoholic families were found to have increased D2R expression^40^. Here, we provide novel and significant evidence to the beginning of the hypodopaminergic hypothesis by identifying intrinsic physiological alterations in VTA dopamine neurons that mediate intrastrain individual differences in alcohol drinking behaviors in an alcohol drinking model.

A key question in studying individual differences is whether these behaviors are innate to the population or are they a result of experience-induced adaptations. This is particularly interesting when studying individual differences in isogenic animal models^41,42^. Epigenetic modifications during early life could alter neural activity to ultimately predispose certain populations to either high or low alcohol drinking behaviors. However, because we observed distributed initial preferences in alcohol drinking behaviors across the alcohol drinking paradigm and because there is little variability in neuronal firing in our EtOH naive group, the possibility of adaptations across the alcohol drinking paradigm between the alcohol drinking groups could underlie the neurophysiological findings we observed. Nevertheless, recent advances in chronic monitoring of genetically defined neural populations will help further elucidate this interesting question.

The use of optogenetics to investigate alcohol drinking behaviors provides the field with the potential to help identify circuits for future therapeutics. However, there is great diversity in the stimulation patterns, animal paradigms, and cell types in which optogenetic techniques are employed. For instance, a separate team of researchers determined that tonic (5 Hz), but not phasic (50 Hz), stimulation of VTA dopamine neurons in rats decreased alcohol drinking behaviors during an EtOH self-administration task^26^. The work done by these investigators, which provided enormous insight into the real time dopamine release dynamics in rats during an EtOH self-administration task, differs from the work presented here in both the optical stimulation protocol and behaviors assessed. Here, by using optical stimulation bursts, we identified the VTA, specifically the VTA-NAc pathway, as a critical mediator of individual differences in alcohol consumption in isogenic C57BL/6J mice. The use of different protocols makes the careful interpretation of results between investigations critical. Additionally, our finding that burst stimulation, but not low or high frequency tonic stimulation, alters alcohol drinking behaviors demonstrates a pattern-specific induction of low alcohol drinking behaviors. Our burst stimulation protocol induces bursts at a higher inter-event frequency than what was observed physiologically in vivo in low alcohol drinking mice. Nonetheless low alcohol drinking mice displayed an increased frequency of burst (+0.5 Hz) compared to the high alcohol drinking mice, suggesting that our stimulation, one burst stimulation every 10 s, may increase the frequency of burst by 0.1 Hz only. While low alcohol drinking mice are in a burst mode an average of 60% of the time, this is still significantly higher than what was observed in EtOH naive and high alcohol drinking mice.

Furthermore, it is critical to validate transgenic mouse lines thoroughly to ensure proper interpretation of results. Non-specific colocalization of Cre-inducible genes in TH^+^ populations was recently discovered in TH::IRES-Cre lines^43^. Many factors could induce this effect, including problems with the transgenic mouse line or problems with viral vectors used^44^. The *TH-BAC-Cre* mouse line we used here has been previously validated to have selective expression of Cre-inducible genes to TH^+^ populations^23–25^. Consistent with previous reports, we discovered similar cell-specific expression level of Cre in our *TH-BAC-Cre* line.

In our investigations, a single 15-min phasic stimulation of VTA dopamine neurons or VTA-NAc neurons reduced alcohol drinking behaviors 48 and 24 h post stimulation, respectively. Previously, we have demonstrated that a 5 min optogenetic phasic stimulation of VTA and VTA-NAc neurons during a social interaction test in mice that received a sub-threshold social-stress induced not only a rapid reduction in social behavior^27,45^, but a reduction in sucrose preference that at least 12 h after the stimulation^27,45^. The same brief optical stimulation physiologically induces a long-lasting increase in the intrinsic excitability of VTA dopamine neurons^27^. A subsequent study discovered that this phasic induction of depressive-like phenotypes also induced an increase in brain derived neurotrophic factor (BDNF) in the NAc in the 5 min phasically stimulated population of mice that received stress specifically, and not in stressed mice that received 5 min of 0.5 Hz tonic stimulation or stress-naive mice that received 5 min phasic stimulation^45^. It should be noticed that the BDNF measure was performed 24 h post-optogenetic stimulation, indicating that a brief phasic stimulation of VTA dopamine neurons induces various sustained effects at molecular level. The detailed mechanisms by which the transient phasic stimulation of VTA dopamine and VTA-NAc neurons induce long-lasting effects on alcohol drinking behaviors in the high alcohol drinking population has yet to be elucidated and are an avenue we hope to explore in the future.

We observed that while VTA dopamine neuron phasic stimulation causes a reduction in alcohol drinking that lasts up to 48 h, phasically stimulating the VTA-NAc population only reduced alcohol drinking behaviors for 24 h. This could implicate another VTA neural circuit that is overactive in low alcohol drinking mice that, in conjunction with VTA-NAc neurons, drives long-lasting reductions in alcohol drinking behaviors. Moreover, immunohistochemical cell counts show that only ~87% of VTA-NAc ChR2-eYFP-infected neurons expressed TH. Recent research has identified independent VTA GABAergic and glutamatergic projecting populations to the NAc, along with dopaminergic projections co-releasing GABA and glutamate^10^. Future investigations to identify the physiological properties of these non-dopaminergic populations would supplement our findings here, to further elucidate the mechanisms of specific alcohol drinking behaviors.

Targeted neural circuit investigations into the causes of excessive alcohol drinking are an emerging focus for many alcohol researchers. Here, we identify a neural circuit to focus future investigations on what causes individual differences in alcohol drinking. This is especially relevant to the alcohol abuse field because of the incredible individual variability in alcohol drinking behaviors.

## Methods

### Animals

Male 6–7-week-old C57BL/6J mice (Jackson laboratories) were ordered group-housed. The *TH-BAC-Cre* (GENSTAT) colony^22^, backcrossed to C57BL/6J line, was managed at Icahn School of Medicine’s Center for Comparative Medicine and Surgery Vivarium and used as experimental subjects beginning at 7–8 weeks of age. Mice were housed on a 12-h light cycle (7:00–19:00), fed standard mouse chow ad libitum and kept at 22–25 °C. All experiments were conducted in accordance with the guidelines of the Institutional Animal Care and Use Committees at Icahn School of Medicine at Mount Sinai.

### Alcohol drinking paradigm

At 7–8 weeks of age, mice were singly housed in non-ventilated standardized Plexiglas mouse cages with corncob bedding and nesting material. On the first day of single-housing, mice were exposed to two 50 mL conical tubes (Fisher Scientific) filled with water and fitted with rubber stoppers with a metal ball bearing sipper tube (Ancare Corporation) for up to 4 days of acclimation. Following acclimation, the experimental mice were weighed. Then one conical tube was filled with a 3% EtOH (Sigma) v/v solution, weighed, and placed next to the subsequently weighed water conical tube for continuous access. Bottle tubes were measured every day and interchanged every day to prevent side preference. After 4 days of exposure to a choice between 3% EtOH v/v and water, the mice were weighed and the 3% EtOH v/v solution was replaced with 6% EtOH v/v, the water replaced, both tubes were weighed and placed in the cage. After 4 days, the mice were weighed and the 6% EtOH v/v solution was replaced with 10% EtOH v/v for a choice that lasted 4 days. On the fourth day of access to 10% EtOH v/v and water, individual alcohol drinking behaviors were determined. Mice were maintained on a choice of 10% EtOH v/v and water until killing. Tubes were refilled accordingly and bottle sides were switched every 2 days once alcohol drinking behaviors were determined. After determining alcohol drinking behaviors, bottle measurements occurred every 4 days. Food was provided ad libitum throughout the alcohol drinking paradigm. EtOH naive mice (control) were also housed in the same conditions, but two tubes of water remained in their cage until day of experiment.

EtOH preference (%) was determined as ((EtOH intake/total fluid intake) × 100). EtOH consumed was determined as g of EtOH according to its concentration/mouse weight across a 24-h or 3-h period. Low alcohol drinking mice were found to have an EtOH preference of <40% and consume <10 g/kg EtOH over 24 h (g/kg per 24 h). High alcohol drinking mice were found to have both an EtOH preference of >60% and consume >10 g/kg EtOH over 24 h (g/kg per 24 h). Mice that did not meet these criteria (middle alcohol drinking mice, ~10% of the population) were not used in the experiments. If alcohol drinking behaviors changed (high alcohol drinkers fell below 60% EtOH preference and 10 mg/kg per 24 h consumption; low alcohol drinkers rose above 40% EtOH preference and 10 mg/kg per 24 h consumption) after the initial 12-day alcohol drinking paradigm on more than two measurement days, mice were excluded from further experiments. Additionally, mice that altered their drinking behaviors following surgeries (high alcohol drinkers fell below 60% EtOH preference and 10 mg/kg per 24 h consumption; low alcohol drinkers rose above 40% EtOH preference and 10 mg/kg per 24 h consumption on average 10% of the population) for >2 days were excluded. Drinking behaviors are plotted in non-overlapping time points. Pre-stimulation preference refers to the 24 h before optogenetic stimulation. Three hours post stimulation time point refers to a 3 h window following the end of optical stimulation (1700–2000 h).

### Preference for a bitter solution

Experimenter was blinded to the alcohol drinking groups until after quinine preferences were determined. Following an alcohol drinking paradigm, a cohort of C57BL/6J high alcohol drinking and low alcohol drinking mice were subjected to a quinine preference test where, in their home cage, the EtOH solution filled tube was replaced with 0.1 mM of quinine, weighed, and placed next to the water filled tube for 24 h. Quinine preference (%) was determined as ((intake of quinine solution/total fluid intake) × 100).

### Blood ethanol concentration

Experimenters were blinded to the alcohol drinking groups until after spectrophotometric data and calculations were performed. At least 300 μL of blood was drawn at 0700 (end of dark cycle) from low and high alcohol drinking mice using a submandibular method. Blood was allowed to sit at room temperature for 1 h and centrifuged at 2500 r.c.f. at 4 °C to separate plasma and erythrocytes. Plasma was collected and stored at −20 °C until day of experiment. Blood EtOH was measured using a ultraviolet spectrophotometer-based alcohol assay kit (Pointe Scientific), and the BEC was determined using an EtOH standard curve measured under the same condition.

### In vivo electrophysiology and analysis

Experimenters performing the procedures and analyses were blinded to the experimental group until after analyses of in vivo spontaneous activity^20,25^. EtOH naive, low alcohol drinking, and high alcohol drinking mice were removed from their drinking cage within 3 h after the dark cycle ended and were anesthetized with 8% chloral hydrate (Sigma-Aldrich, 400 mg/kg, ip) until fully sedated. Mice were head-fixed to a stereotaxic frame (Kopf), and under aseptic conditions, the skull was exposed to reveal bregma and flat-tested. Ophthalmic ointment (Lubrifresh P.M., Fisher Hamilton Scientific) was applied to prevent eyes from drying and body temperature was maintained using a regulated heat pad. Coordinates for the VTA were obtained from Bregma (anterior/posterior, −2.92/−3.88; medial/lateral ± 0.24/0.96; and DV = −3.5/−4.5) and a cranial window was created above the VTA. A small hole for a reference wire was drilled anterior to bregma. Glass electrodes filled with 2 M NaCl were lowered slowly using a micromanipulator (Sutter Instruments DP-311). Signals were amplified (Differential Amplifier) and filtered (0.3–1 kHz band-pass). Voltage data were acquired using an Axon Digidata Data Acquisition System, sampled using 16-bit resolution at 32 kHz, and stored using pCLAMP for further offline analysis.

Single-unit, juxtacellular in vivo recordings were performed as previously described^20,21,25^. Only the firing activity of putative VTA dopamine neurons was analyzed. Neurons were identified to be putative VTA dopamine neurons before recording using standardized electrophysiological criteria including a triphasic waveform with set filters, action potential duration from start to negative trough >1.1 ms, slow firing rate (≤10 spikes per s). Bursts identified during analysis and occurred when two spikes within <80 ms and offset set when no activity occurred for >160 ms^7,20,21,46^. Spike times were measured using Clampfit 10.2 and data were further processed using *R* software as previously described^21,46,47^. Briefly, activity was quantified by overlapping 60 s windows shifted every 15 s. Frequency of bursting is defined by the average amount of bursts. Percentage of SWBs corresponds to the percentage of spikes discharged within a burst throughout the recording period. Finally, burst length was determined after measuring the number of spikes within a burst and averaged per recorded neuron.

### Viral constructs

For expression of blue-light activated channels or control fluorophores in VTA dopamine neurons of *TH-BAC-Cre* mice, AAV5-EF1a-DIO-hChR2(H134R)-eYFP/mCherry or AAV5-EF1a-DIO-eYFP/mCherry for injection into the VTA was obtained from the Gene Therapy Center Vector Core at University of North Carolina, Chapel Hill.

For expression of blue-light activated channels or control fluorophores in VTA-NAc or VTA-mPFC neurons of C57BL/6J mice, we used a dual viral system using (1) retrograding AAV2/5-CMV-HI-Cre-WPRE-SV40 obtained from the Penn Vector Core Gene Therapy Program at Perelman School of Medicine at University of Pennsylvania for bilateral injection into the NAc or mPFC and (2) AAV5-EF1a-DIO-hChR2(H134R)-eYFP or AAV5-EF1a-DIO-eYFP for injection into the VTA obtained from the Gene Therapy Center Vector Core at University of North Carolina, Chapel Hill.

For pathway-specific in vitro electrophysiological recordings of VTA-NAc, green retrograding microbeads (Lumafluor) were obtained from Lumafluor Inc. for bilateral injection into the NAc.

For expression of viral fluorophores in VTA-NAc or VTA-mPFC dopamine neurons of *TH-BAC-Cre* mice for in vitro electrophysiological recordings, retrograding AAV2/5-EF1a-DIO-eYFP-WPRE-hGH (Addgene 27056) was obtained from the Penn Vector Core Gene Therapy Program at Perelman School of Medicine at University of Pennsylvania for bilateral injection into the NAc or mPFC.

### Stereotaxic surgeries and optic fiber implantation

EtOH naive group-housed *TH-BAC-Cre* or C57BL/6J mice at 7 weeks of age were anesthetized with ketamine (100 mg/kg)/xylazine (10 mg/kg) and head-fixed to a stereotaxic frame (Kopf)). Ophthalmic ointment was applied to prevent eyes from drying and body temperature was maintained using regulated heated lamps. For bilateral virus or microbead injections (0.3–0.5 μL) under aseptic conditions, bregma was exposed, the head was flat-tested, and coordinates for VTA (anterior/posterior, −3.3 mm; medial/lateral, +0.75 mm; dorsal/ventral, −4.6 mm; 7° angle), NAc (anterior/posterior, 1.4 mm; medial/lateral, +1.5 mm; dorsal/ventral, −4.3 mm; 10° angle), or mPFC (anterior/posterior, +1.7 mm; medial/lateral, +0.75 mm; dorsal/ventral, −2.5 mm; 15° angle) were calculated.

Mice used for VTA dopaminergic optogenetic manipulations were randomly selected to receive either AAV5-EF1a-DIO-hChR2(H134R)-eYFP or AAV5-EF1a-DIO- eYFP into the VTA before the alcohol drinking paradigm.

Mice used for pathway-specific VTA-NAc or VTA-mPFC optogenetic stimulations received AAV2/5-CMV-HI-Cre-WPRE-SV40 into the NAc or mPFC and were randomly selected to receive either AAV5-EF1a-DIO-hChR2(H134R)-eYFP or AAV5-EF1a-DIO- eYFP into the VTA before the alcohol drinking paradigm.

For VTA-NAc projection-specific in vitro recordings, C57BL6J mice received retrograding microbeads. For VTA-NAc or VTA-mPFC projection- and dopaminergic-specific in vitro recordings, *TH-BAC-Cre* mice received retrograding AAV2/5-EF1α-DIO-eYFP-WPRE-hGH injected into the NAc or mPFC before the alcohol drinking paradigm.

After all injections, skin was sutured shut and antibacterial Neosporin with topical pain relief was applied to the wound. Immediately following the surgeries, mice were singly housed in cages described in the alcohol drinking paradigm section with two 50 mL conical tubes filled with water and fitted with rubber stoppers with sipping tubes for acclimation for 1 week. Mice were then randomly selected to remain EtOH naive, or partake in the alcohol drinking paradigm.

For bilateral optogenetic stimulations of VTA dopamine, VTA-NAc, or VTA-mPFC neurons, high alcohol drinking or EtOH naive mice with either AAV5-DIO-ChR2-eYFP or AAV5-DIO-eYFP were selected after the alcohol drinking paradigm to receive a bilateral chronic fiber implantation above the VTA. A skull screw was first implanted anterior to bregma and then VTA coordinates for fiber placement were obtained (anterior/posterior, −3.3 medial/lateral, +1.0; dorsal/ventral, −4.4; 7° angle). Implants were cemented secure with dental acrylic (Grip Cement Dentsply). Immediately following these described surgeries, mice were placed back into their home cages to continue the alcohol drinking paradigm section with two 50 mL conical tubes filled with water or 10% EtOH for 1 week before optogenetic manipulations. Mice that did not display their high alcohol drinking behaviors after surgery were not used for behavioral optogenetic manipulations (see exclusion criteria under alcohol drinking paradigm).

### Optogenetic manipulation of VTA neurons

Chronic implantable optic fibers were constructed in the laboratory using ceramic stick ferrules (1.25 mm, Precision Fiber Products) and stripped 200 µm optic fibers (Thor laboratories). Optical fiber patch cords were also constructed in the laboratory using 200 µm thick optic fibers up to 2 m long. Using FC/PC adaptors, patch cords were attached to a laser splitter which was connected to a 100 mW 473-nm blue laser (OEM Laser Systems, BL-473–00–100-CWM-SD05-LED-0). Light pulses were generated using a function generator (Agilent Technologies, 33220 A). Before implantation, chronic implantable optic fibers were tested using a laser power meter with a slim head photodiode sensor head to ensure phasic stimulation protocol emitted a light power that was between 5 and 10 mW (Thor Laboratories, PM200 and S130A).

Experimenter was blinded to the stimulation group (eYFP or ChR2) prior to stimulation and during post-stimulation two-bottle choice measurements. Twenty-four hours before optical stimulation, alcohol drinking behaviors were determined (pre-stim). Both eYFP and ChR2 high alcohol drinking groups that had optic ferrule fibers were connected to the laser using patch cords to allow for free movement in their home cage. For phasic stimulation of VTA dopamine neurons in high alcohol drinking and EtOH naive mice, as well as VTA-NAc and VTA-mPFC neurons in high alcohol drinking mice, we used a phasic (burst) stimulation protocol physiologically observed in vivo in VTA dopamine neurons of low alcohol drinking mice (5 spikes, 40 ms pulse width, 10 ms spike interval, 20 Hz) every 10 s for 15 min. This stimulation protocol has been previously shown to have high spike fidelity in vivo^27^, induce optically elicited spikes and currents in vitro (Figs. 3 and 5, see also ref. ^23^), and the length of time has been demonstrated to induce alterations in VTA-mediated social behaviors^23^. Tonic stimulation of 0.5  Hz OF VTA dopamine neurons to control for induction of five spikes was performed in VTA DA neurons of high alcohol drinking mice based on a previously published (5 spikes, 15 ms pulse width, 0.5 Hz, 1.985 s inter-spike interval, 10 s period^27^). Continuous, 5 Hz stimulation for 15 min was also performed according to a previous study (250 spikes, 4 ms pulse width, 5 Hz, 196 ms inter-spike interval^26^) to further characterize pattern-driven alcohol drinking behaviors. eYFP high alcohol drinking groups received this blue-light stimulation also to control for handling and laser conditions. There was no access to food, water, or alcohol during the 15 min of stimulation. Optogenetic stimulations occurred from 1600 to 1700 h. Mice were weighed following optical stimulation, and by 1700 h, the alcohol drinking paradigm resumed to collect alcohol drinking data. Measurements of drinking bottles were performed as carefully and quickly as possible and did not take >2 min per mouse.

### In vitro electrophysiology and analysis

Experimenter was double blinded until after analyses of spontaneous activity and excitability data. For circuit-specific recordings from VTA-NAc neurons of EtOH naive, low alcohol drinking and high alcohol drinking mice, whole-cell recordings were obtained from C57BL/6J mice that had been injected with retrograding microbeads into the NAc. For projection- and cell-specific recordings from VTA-NAc or VTA-mPFC dopamine neurons of EtOH naive, low alcohol drinking and high alcohol drinking mice, whole-cell recordings were obtained from *TH-BAC-Cre* mice that had been injected with retrograding AAV2/5-EF1a-DIO-eYFP-WPRE-hGH in the NAc or mPFC.

EtOH naive mice, low alcohol drinking mice, and high alcohol drinking mice were anesthetized with isoflurane and perfused immediately with oxygenated, ice-cold NaCl aCSF for 60 s. NaCl aCSF was composed of 128 mM NaCl, 3 mM KCl, 1.25 mM NaH_2_PO_4_, 10 mM d-glucose, 24 mM NaHCO_3_, 2 mM CaCl_2_ and 2 mM MgCl_2_, pH 7.4, 295–305 mOsm. The brain was removed quickly and placed into a NaCl-depleted, oxygenated sucrose aCSF (254 mM sucrose, 3 mM KCl, 1.25 mM NaH_2_PO4, 10 mM d-glucose, 24 mM NaHCO_3_, 2 mM CaCl_2_ and 2 mM MgCl_2_, pH 7.4, 295–305 mOsm) solution for 1 min. The brain was blocked, superglued to a vibratome (DTK-1000, Ted Pella) slicing dish, and covered with oxygenated sucrose aCSF. The VTA was obtained at 250 µm slices and immediately transferred to a heated (37 °C) recovery chamber filled with NaCl aCSF for 1 h.

The VTA was identified by its location using infrared differential interference contrast microscopy (Olympus). Patch pipettes (3–5 mΩ) for whole-cell voltage-clamp and current-clamp recordings of Lumafluor (+) VTA-NAc neurons or eYFP(+) VTA-NAc or VTA-mPFC dopamine neurons were filled with internal solution containing the following: 115 mM potassium gluconate, 20 mM KCl, 1.5 mM MgCl_2_, 10 mM phosphocreatine, 10 mM HEPES, 2 mM magnesium ATP and 0.5 mM GTP, pH 7.2, 285 mOsm. Whole-cell recordings were carried out using aCSF at 32 °C (flow rate = 2.5 mL/min). After obtaining whole-cell access to neurons, resting membrane potential and spontaneous action potentials were recorded in current clamp (*I* = 0) using a Molecular Devices 700B amplifier, digitized with Digidata 1440A, and acquired with pCLAMP 10 (Molecular Devices). Location of recorded cells was recorded in recording notebook. Hyperpolarization-activated currents (*I*_h_) were measured in voltage-clamp mode (VC, holding potential at −60 mV) by conducting a series of 3 s voltage injections of 10 mV incremental steps to attain membrane voltages from −120 to −60 mV. Cell excitability was measured with 2 s incremental steps of current injections (0, 50, 100, and 150 pA) in current-clamp mode. Series resistance was monitored during all recordings with changes in resistance +10% qualifying the cell for exclusion.

Monitoring of firing rate during EtOH bath application (20, 50, and 100 mM EtOH in aCSF) was performed under a partial access mode once a giga seal was obtained on the cell with patch pipettes of (3–5 mΩ) filled with internal solution listed above. Partial access to the cell has been shown to reliably maintain access for long recordings while preventing run down and with less disturbance to the neuronal milieu^48^.

Analyses of in vitro electrophysiological data were performed offline using Clampfit.

### Immunohistochemistry

Mice were quickly anesthetized with urethane and perfused with 20 mL of cold 1× PBS (OmniPure, Bio-Rad) followed by 30 mL of cold 4% PFA (Fisher Hamilton Scientific). Brains were removed and allowed to post-fix in PFA at 4 °C for 24 h before being submerged in a 30% sucrose solution and kept at 4 °C. Once the brains had sunk in sucrose, it was washed quickly with 1× PBS with sodium azide (Sigma-Aldrich) and mounted on a freezing microtome for sectioning at 35 µm. Tissue slices were kept in 1× PBS with sodium azide until day of immunolabeling.

Sections were washed three times for 10 min in 1× PBS, then blocked in 2% bovine serum albumin (Sigma-Aldrich) in 1× PBS with 0.3% Triton-X (Sigma-Aldrich) for 1 h. All antibodies were diluted in 2% bovine serum albumin in 1× PBS with 0.3% Triton-X. Tissue was incubated with primary antibodies for anti-TH grown in mouse (1:10,000, T2928, Sigma-Aldrich) and anti-GFP grown in rabbit (1:2000, A-6455, Invitrogen) overnight at 4 °C. Sections were then washed three times with 1× PBS with 0.3% Triton-X. Tissue was blocked in secondary antibodies for anti-mouse Cy5/Alexa-647 (1:500, 715605150, Jackson Immuno) and anti-rabbit Cy2/Alexa-488 (1:500, 715545152, Jackson Immuno) for 1 h at room temperature. Sections were washed three times with 1× PBS for 10 min and mounted on slides with ProLong Gold antifade reagent with DAPI (P36931, Life Technologies). Slides were stored at −20 °C until imaging. *Z*-stacked images were acquired with a Zeiss LSM780 multi photon confocal system and *z*-stacks were compressed using ImageJ. Cell counts were obtained using the Cell Counter plug-in on ImageJ.

### Statistical analysis

Graph Pad Prism (version 6, La Jolla, CA, USA) or *R* was used to statistically analyze data sets. Graphs were created using Graph Pad Prism once groups were unblinded. If data were not normally distributed, non-parametric analyses were performed. Alcohol drinking behaviors, total liquid intake, and body weights data were analyzed using a two-way repeated measures (RM) analysis of variance (ANOVA) when subject (i.e., matching) effects were significant; if the subject effects were not significant, a two-way ANOVA was performed. ANOVA analyses were followed by a Bonferroni post hoc test when a group effect (eYFP vs ChR2) or interactions between factors were observed. Alcohol drinking behaviors and total liquid intake at 24 h pre-, and 24, 48, and 72 h post-optogenetic stimulation time points were analyzed using ANOVA analysis in which the post-stimulation time points represent 24 h, non-overlapping blocks. Unpaired, two-tailed Student’s *t* tests were used for 3 h time points. Quinine preference and BEC data were analyzed using unpaired two-tailed Student’s *t* tests. *I*_h_. VTA-NAc dopamine neuron excitability and EtOH responsivity data were analyzed using a two-way RM ANOVA. Data for in vivo electrophysiology were analyzed using a Kruskal–Wallis *χ*^2^-test because of non-normal distributions followed by Dunn’s multiple comparison test. VTA-NAc and VTA-mPFC dopamine neuron spontaneous firing rate (spontaneous activity) was analyzed using a one-way ANOVA followed by a Bonferroni post hoc test. Correlational analyses were used to average in vivo VTA dopamine neuron firing rate or in vitro VTA-NAc dopamine neuron firing with alcohol drinking behaviors. *P* values <0.05 were considered to be statistically significant for all data.

### Data availability

The authors declare that the data supporting the findings of this study are available within the paper and its supplementary information files or are available from the corresponding author upon request.

## Electronic supplementary material

Supplementary Information
