# Author Correction: Midbrain circuit regulation of individual alcohol drinking behaviors in mice

**DOI:** 10.1038/s41467-018-02921-w

**Published:** 2018-02-08

**Authors:** Barbara Juarez, Carole Morel, Stacy M. Ku, Yutong Liu, Hongxing Zhang, Sarah Montgomery, Hilledna Gregoire, Efrain Ribeiro, Marshall Crumiller, Ciorana Roman-Ortiz, Jessica J. Walsh, Kelcy Jackson, Denise E. Croote, Yingbo Zhu, Song Zhang, Leandro F. Vendruscolo, Scott Edwards, Amanda Roberts, Georgia E. Hodes, Yongke Lu, Erin S. Calipari, Dipesh Chaudhury, Allyson K. Friedman, Ming-Hu Han

**Affiliations:** 1 Department of Pharmacological Sciences and Institute for Systems Biomedicine, Icahn School of Medicine at Mount Sinai, New York, NY 10029 USA; 2 Fishberg Department of Neuroscience and Friedman Brain Institute, Icahn School of Medicine at Mount Sinai, New York, NY 10029 USA; 3 Jiangsu Province Key Laboratory of Anesthesiology, Xuzhou Medical University, Xuzhou, 221002 Jiangsu China; 4 Intramural Research Program, National Institute on Drug Abuse, National Institutes of Health, Baltimore, MD 21224 USA; 5 Department of Physiology, Alcohol and Drug Abuse Center of Excellence, Neuroscience Center of Excellence, Louisiana State University Health Sciences Center, New Orleans, LA 70112 USA; 6 Department of Molecular and Cellular Neuroscience, The Scripps Research Institute, San Diego, CA 92037 USA; 7 Department of Medicine, Icahn School of Medicine at Mount Sinai, New York, CA 10029 USA; 8 Department of Health Sciences, College of Public Health, East Tennessee State University, Johnson City, TN 37614 USA; 9 Department of Pharmacology, Vanderbilt University, Nashville, TN 37232 USA; 10 Division of Science, New York University Abu Dhabi (NYUAD), Saadiyat Island Campus, Abu Dhabi, PO Box 129188 United Arab Emirates; 11 Department of Biological Sciences, Hunter College, City University of New York, New York, NY 10065 USA

Correction to: *Nature Communications* 10.1038/s41467-017-02365-8, published online: 20 Dec 2017

The original version of this Article contained an error in the spelling of the author Scott Edwards, which was incorrectly given as Scott Edward. This has now been corrected in both the PDF and HTML versions of the Article.

