# Supplementary material for: Midbrain circuit regulation of individual alcohol drinking behaviors in mice
Source: Nat Commun. 2017 Dec 20;8:2220. doi: 10.1038/s41467-017-02365-8 (PMC5738419; doi:10.1038/s41467-017-02365-8)
Supplement: Supplementary file 1 — Supplementary Information [file 41467_2017_2365_MOESM1_ESM.pdf]

SUPPLEMENTARY FIGURES

Supplementary Figure 1

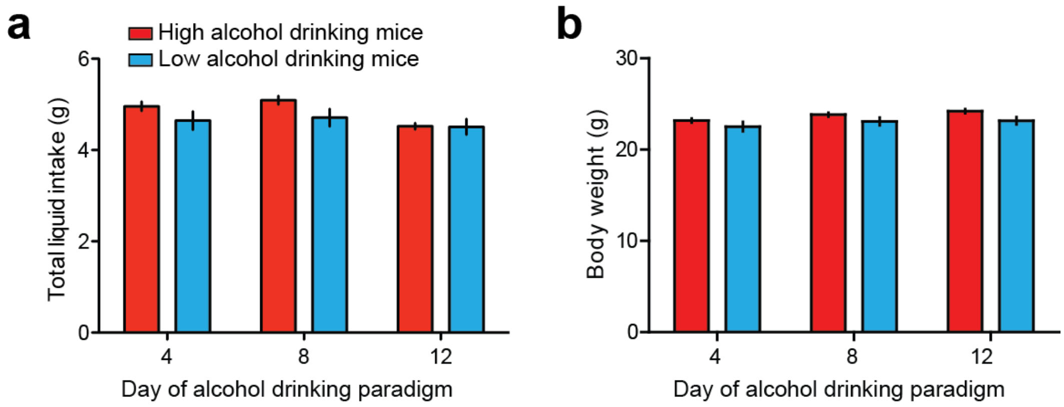

Supplementary Figure 1 Total liquid consumed and body weight did not differ between the alcohol drinking groups (**a**) Total liquid intake (two-way RM ANOVA: interaction effect $F_{(2, 92)}$=2.120, *P*=0.1259; drinking group effect $F_{(1, 92)}$=1.571, *P*=0.2164; Bonferroni post-hoc, ns. *n*=8 mice; *n*=40 mice). (**b**) Body weight (two-way RM ANOVA: interaction effect $F_{(2, 92)}$=1.763, *P*=0.1773; drinking group effect $F_{(1, 92)}$=2.526, *P*=0.1189; Bonferroni post-hoc, ns. *n*=8 mice; *n*=40 mice).

Supplementary Figure 2

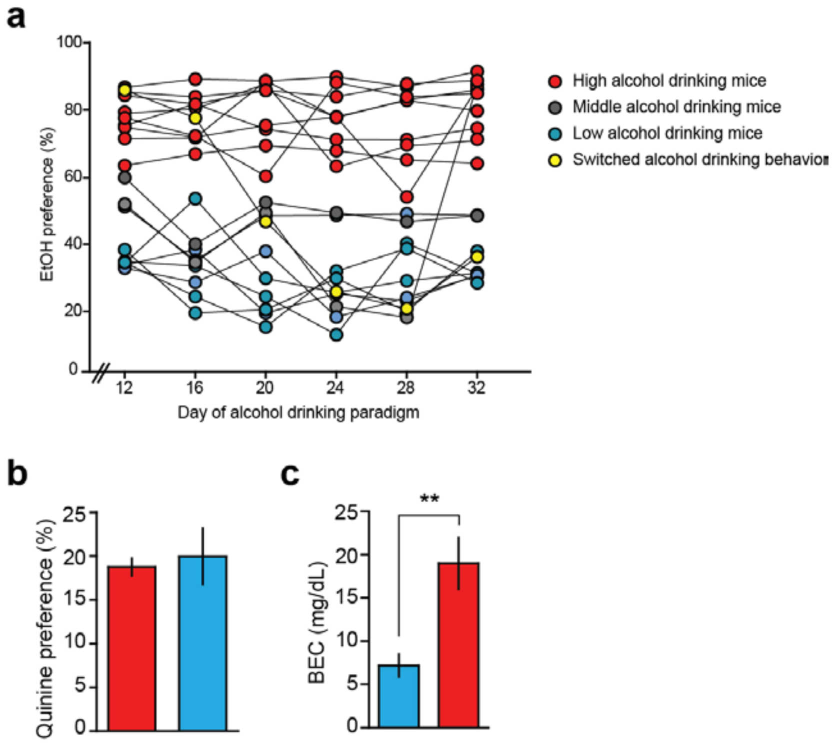

Supplementary Figure 2 EtOH preference persisted across time, quinine preference was not different between groups and blood ethanol concentrations were different. (**a**) Long-term recordings from a cohort of alcohol drinking C57BL/6J mice. Grey data point are middle alcohol drinking mice that are excluded from future investigation. Yellow data point is a mouse that changed its EtOH preference after behavior determination and was excluded. (**b**) Quinine taste discrimination (unpaired, two-tailed t-test $t_{(6)}$=0.3532, P=0.7360. n=4 mice/group). (**c**) Blood ethanol concentrations (unpaired, two-tailed t-test $t_{(8)}$=3.627, $^{**}p$<0.01. n=5 mice/group). Data represented as mean $\pm$ S.E.M.

Supplementary Figure 3

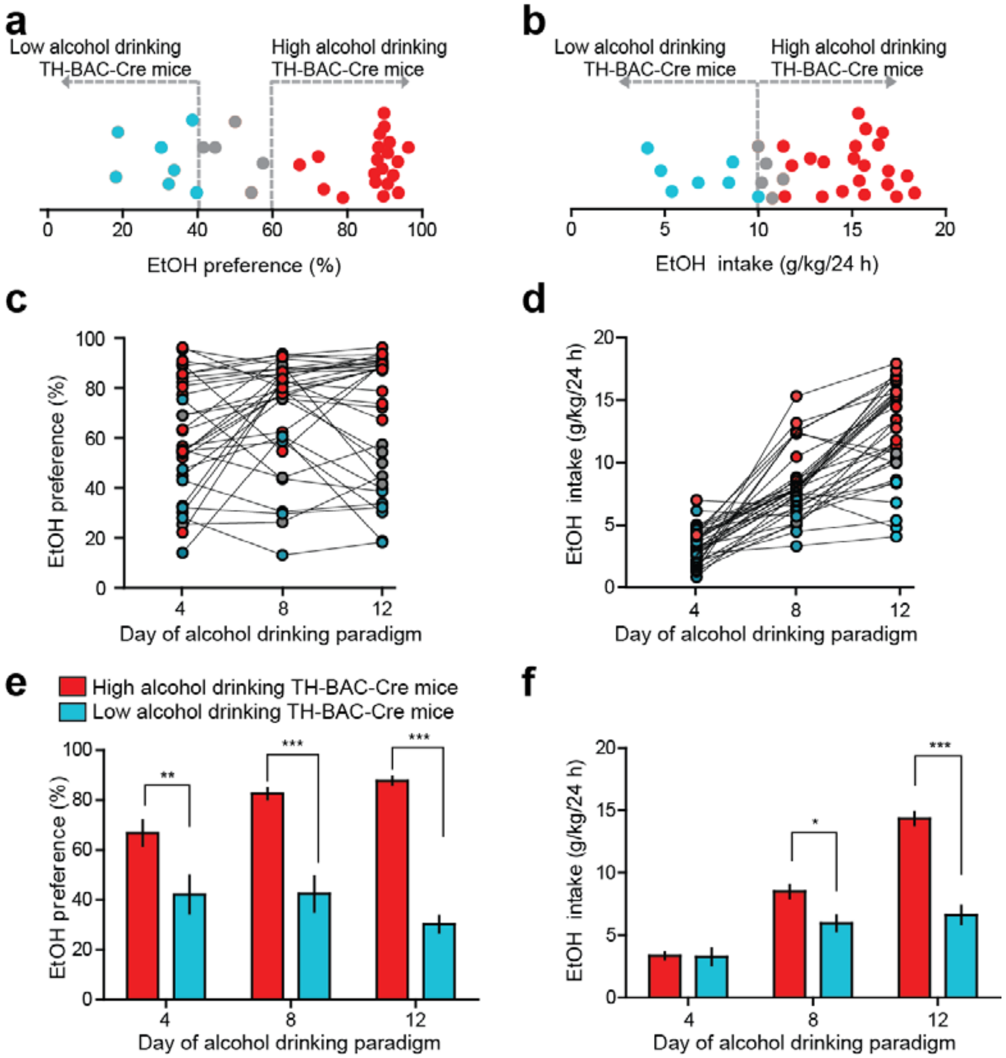

Supplementary Figure 3 TH-BAC-Cre mice backcrossed to C57BL/6J split into alcohol drinking groups. (**a**) Distribution of EtOH preference on 12th day of alcohol drinking paradigm (*n*=33). (**b**) Distribution of EtOH intake on 12th day of alcohol drinking paradigm (*n*=33). (**c**) Individual EtOH preferences plotted across alcohol drinking paradigm (*n*=33). (**d**) Individual EtOH intakes plotted across alcohol drinking paradigm (*n*=33). (**e**) Low and high alcohol drinking group EtOH preferences (two-way RM ANOVA: interaction effect $F_{(2, 50)}$=7.472, ***P*<0.01; drinking group effect $F_{(1, 50)}$=80.49, ****P*<0.001; Bonferroni post-hoc, ***P*<0.01, ****P*<0.001. *n*=7 mice; *n*=20 mice). (**f**) Low and high alcohol drinking group EtOH intake over 24 hrs (two-way RM ANOVA: interaction effect $F_{(2,50)}$=25.26, ****P*<0.001; drinking group effect $F_{(1,50)}$=35.12, ****P*<0.01; Bonferroni post-hoc, **P*<0.05, ****P*<0.001. *n*=7 mice; *n*=20 mice). All data represented as mean ± S.E.M.

Supplementary Figure 4

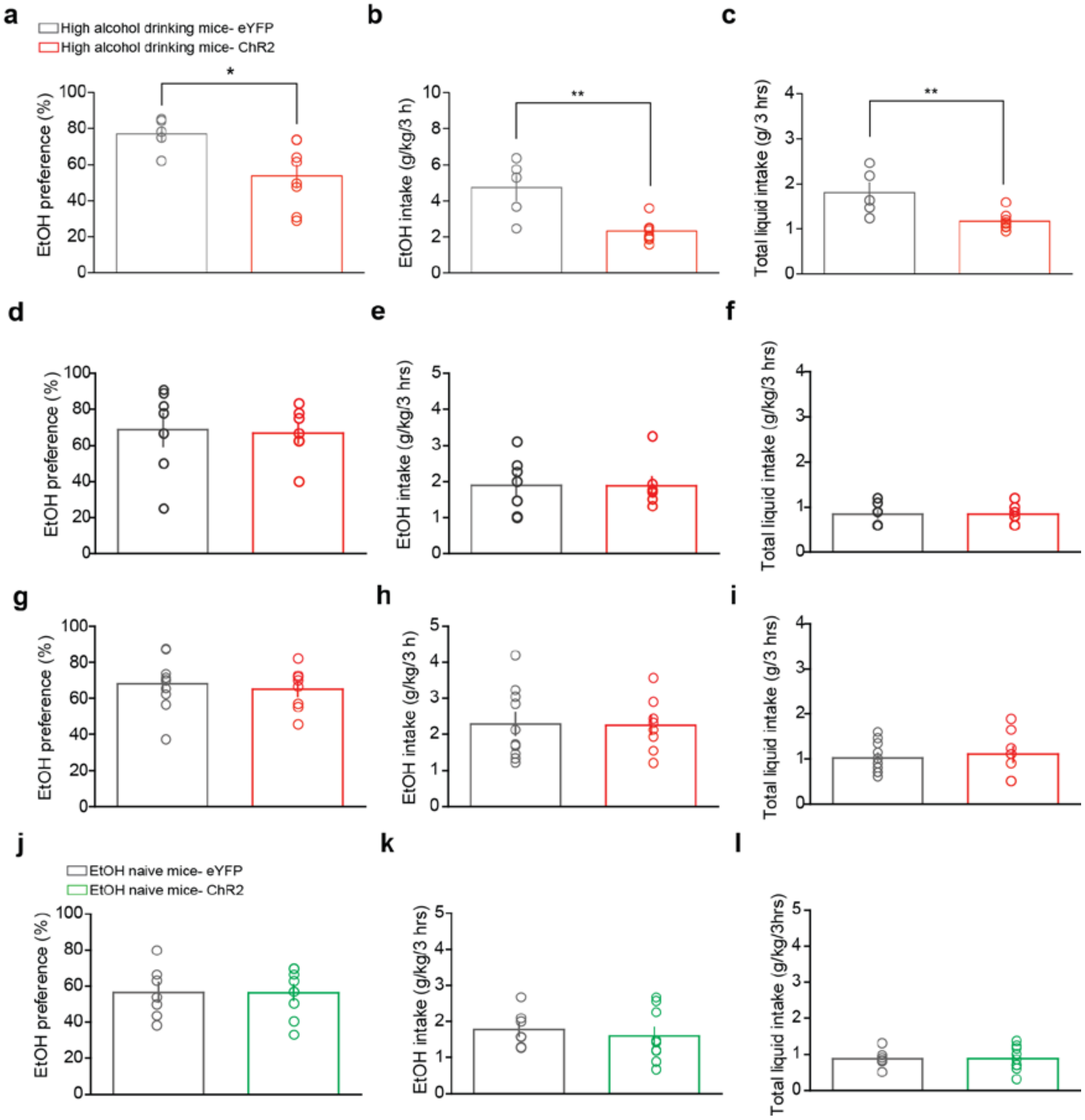

Supplementary Figure 4 Alcohol drinking behaviors of TH-BAC-Cre mice 3 h following 15 minutes of VTA dopamine neuron stimulation (**a**) EtOH preference of high alcohol drinking mice following phasic stimulation (unpaired, two-tailed t-test: $t_{(11)}$=2.696, **P*<0.05. *n*=5, *n*=8 mice). (**b**) EtOH intake of high alcohol drinking mice following phasic stimulation (unpaired, two-tailed t-test, $t_{(11)}$=3.902, ***P*<0.01, *n*=5, *n*=8). (**c**) Total liquid intake of high alcohol drinking mice following phasic stimulation unpaired, two-tailed t-test, $t_{(11)}$=3.187, ***P*<0.01, *n*=5, *n*=8). (**d**) EtOH preference of high alcohol drinking mice following 5 Hz stimulation (unpaired, two-tailed t-test, $t_{(12)}$=0.1799, *P*=0.8602, *n*=7, *n*=7). (**e**) EtOH intake of high alcohol drinking mice following 5 Hz stimulation (unpaired t-test, $t_{(12)}$=0.02702, *P*=0.9789, *n*=7, *n*=7). (**f**) Total liquid intake of high alcohol drinking mice following 5 Hz stimulation (unpaired, two-tailed t-test, $t_{(12)}$=0.0, *P*=1.000, *n*=7, *n*=7). (**g**) EtOH preference of high alcohol drinking mice following 0.5 Hz stimulation (unpaired, two-tailed t-test, $t_{(16)}$=0.4713, *P*=0.6438,

n=10, *n*=8). (**h**) EtOH intake of high alcohol drinking mice following 0.5 Hz stimulation (unpaired t-test, $t_{(16)}$=0.07506, *P*=0.9411, *n*=10, *n*=8). (**i**) Total liquid intake of high alcohol drinking mice following 0.5 Hz stimulation (unpaired, two-tailed t-test, $t_{(16)}$=0.4286, *P*=0.6739, *n*=10, *n*=8). (**j**) EtOH preference of EtOH naïve mice following phasic stimulation (unpaired, two-tailed t-test $t_{(14)}$=0.0171, *P*=0.9866 *n*=7, *n*=9). (**k**) EtOH intake 3 h post phasic stimulation of VTA DA neurons in previously EtOH naïve mice (unpaired, two-tailed t-test $t_{(14)}$=0.5814, *P*=0.5702, *n*=7, *n*=9). (**l**) Total liquid intake of EtOH naïve mice 3 h following phasic stimulation (unpaired, two-tailed t-test, $t_{(14)}$=0.04183, *P*=0.9672, *n*=7, *n*=9). All data represented as mean ± S.E.M.

Supplementary Figure 5

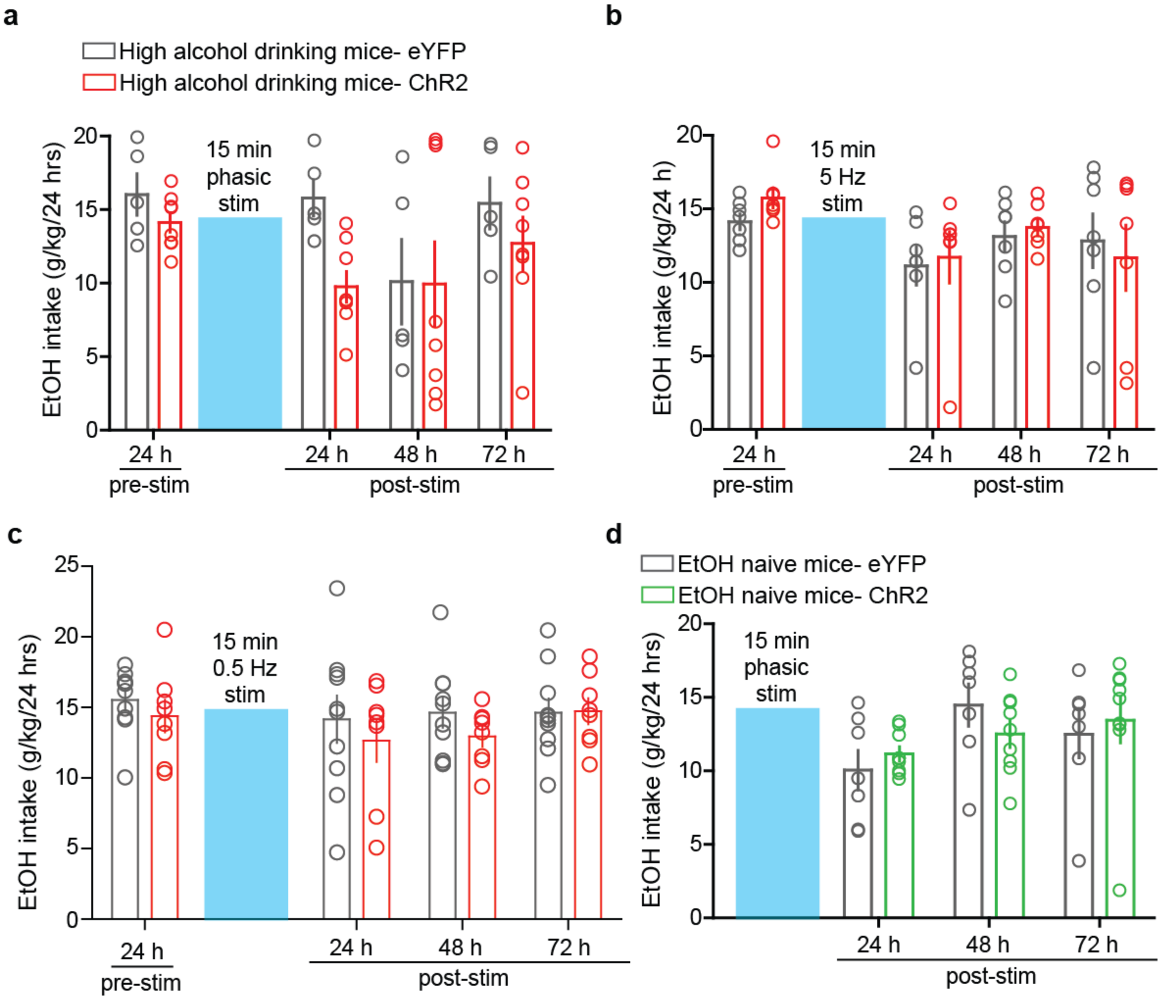

Supplementary Figure 5 EtOH intake behaviors of 15 min VTA dopamine neuron stimulation cohorts. (**a**) EtOH intake of high alcohol drinking mice that received phasic stimulation (two-way RM ANOVA: interaction effect $F_{(3,33)}$=1.741, *P*>0.05; drinking group effect $F_{(1,33)}$=1.487, *P*>0.05; time effect $F_{(3,33)}$=5.473, *P*<0.01; Bonferroni post-hoc test, *P*>0.05, *n*=5 mice; *n*=8 mice). (**b**) EtOH intake of high alcohol drinking mice that received 5 Hz stimulation (two-way RM ANOVA: interaction effect $F_{(3,36)}$=0.4938, *P*>0.05; drinking group effect $F_{(1,36)}$=0.09888, *P*>0.05; time effect $F_{(3,36)}$=3.469, *P*<0.05; Bonferroni post-hoc test, *P*>0.05, *n*=7 mice; *n*=7 mice). (**c**) EtOH intake of high alcohol drinking mice that received 0.5 Hz stimulation (two-way RM ANOVA: interaction effect $F_{(3,48)}$=0.4170, *P*>0.05; drinking group effect $F_{(1,48)}$=0.7353, *P*>0.05; time effect $F_{(3,48)}$=1.348, *P*>0.05; Bonferroni post-hoc test, *P*>0.05, *n*=10 mice; *n*=8 mice). (**d**) EtOH intake of EtOH naïve mice that received phasic stimulation (two-way RM ANOVA: interaction effect $F_{(2,28)}$=1.157, *P*>0.05; drinking group effect $F_{(1,28)}$=0.0009187, *P*>0.05; time effect $F_{(2.28)}$=3.678, **P*<0.05; Bonferroni post-hoc test, *P*>0.05, *n*=7 mice; *n*=9 mice). All data represented as mean $\pm$ S.E.M.

Supplementary Figure 6

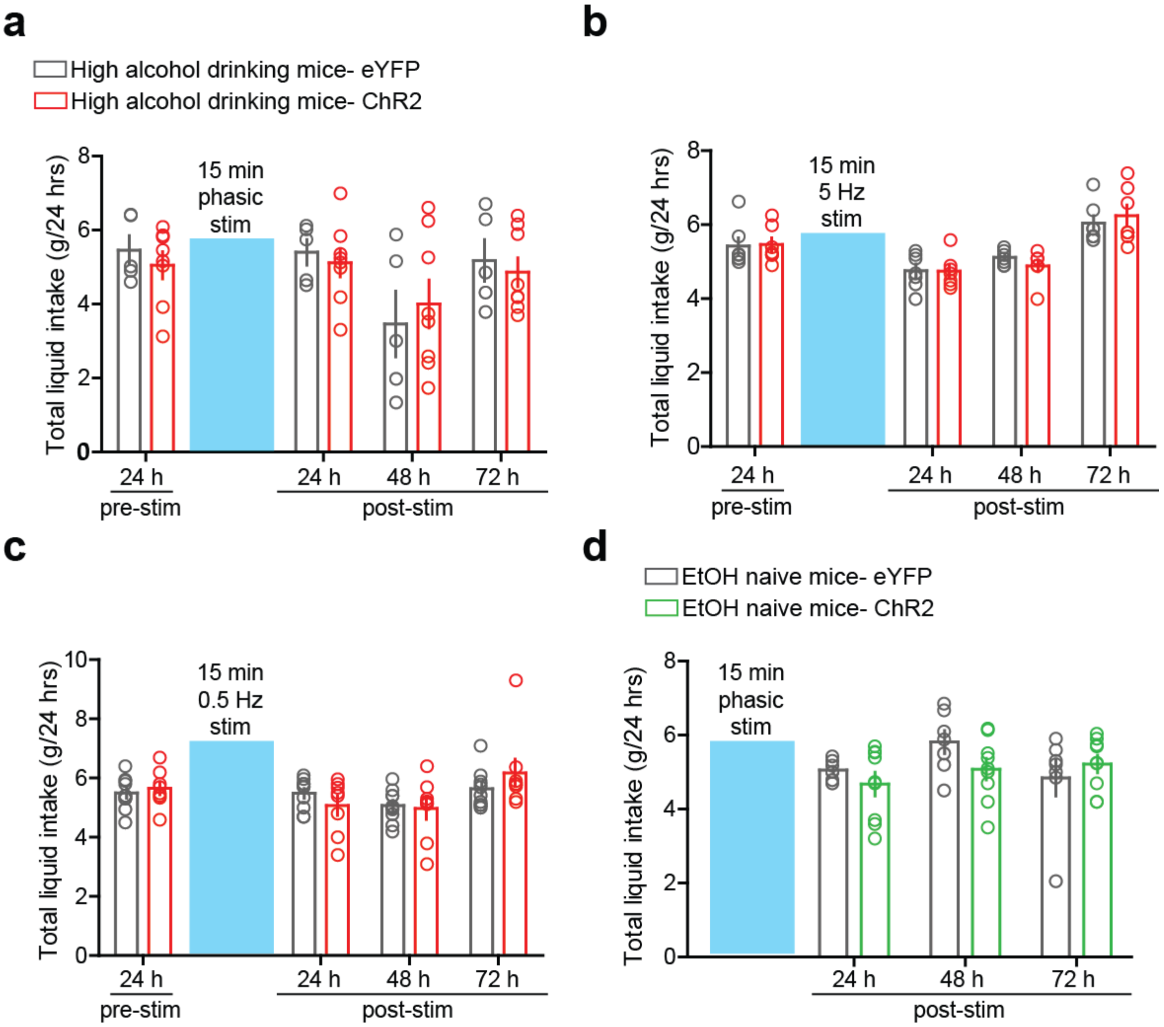

Supplementary Figure 6 Total liquid intake of mice that received VTA DA neuron stimulations. (**a**) Total liquid intake of high alcohol drinking mice that received phasic stimulation (two-way RM ANOVA: interaction effect $F_{(3,33)}=0.7014$, $P>0.05$; drinking group effect $F_{(1,33)}=0.03959$, $P>0.05$; time effect $F_{(3,33)}=7.665$, $P<0.001$; Bonferroni post-hoc test, $P>0.05$, $n=5$ mice; $n=8$ mice). (**b**) Total liquid intake of high alcohol drinking mice that received 5 Hz stimulation (two-way ANOVA: interaction effect $F_{(3,48)}=0.\ 7351$, $P>0.05$; drinking group effect $F_{(1,36)}=0.0.00002774$, $P>0.05$; time effect $F_{(3,36)}=20.28$, $P<0.0001$; Bonferroni post-hoc test, $P>0.05$, $n=7$ mice; $n=7$ mice). (**c**) Total liquid intake of high alcohol drinking mice that received 0.5 Hz stimulation (two-way ANOVA: interaction effect $F_{(3,48)}=1.168$, $P>0.05$; drinking group effect $F_{(1,48)}=0.05518$, $P>0.05$; time effect $F_{(3,48)}=4.154$, $P<0.01$; Bonferroni post-hoc test, $P>0.05$, $n=10$ mice; $n=8$ mice). (**d**) Total liquid intake of EtOH naïve mice that received phasic stimulation (two-way RM ANOVA: interaction effect $F_{(2,28)}=2.572$, $P>0.05$; drinking group effect $F_{(1,28)}=0.5556$, $P>0.05$; time effect $F_{(2.28)}=2.868$, $P<0.05$; Bonferroni post-hoc test, $P>0.05$, $n=7$ mice; $n=9$ mice). All data represented as mean $\pm$ S.E.M.

Supplementary Figure 7

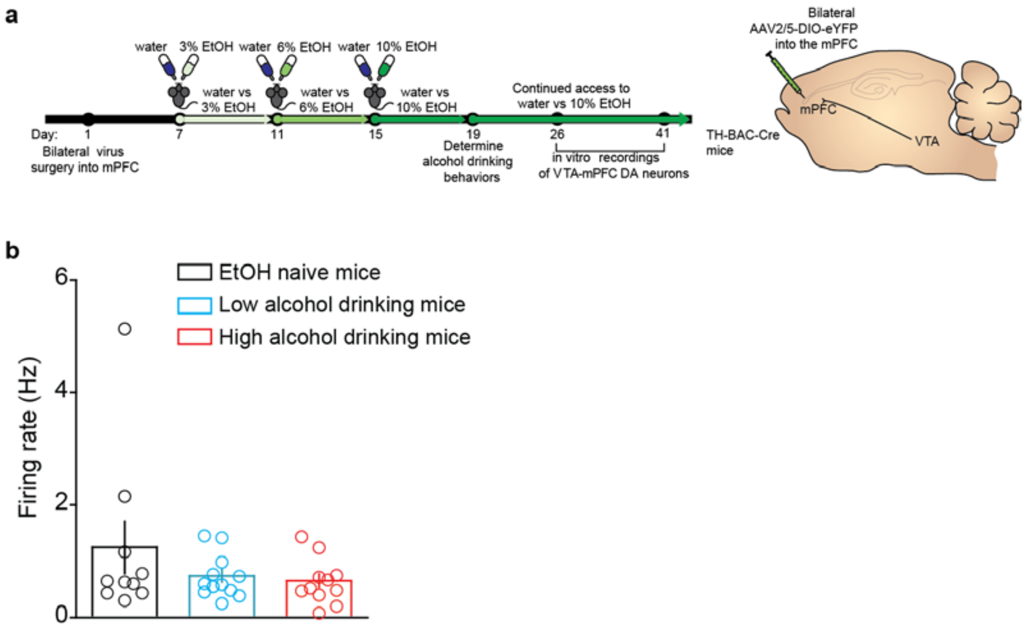

Supplementary Figure 7 There were no differences in firing rate in VTA-mPFC dopamine neurons. (**a**) Timeline of circuit dissecting electrophysiological experiments with schematic of surgeries. (**b**) VTA-mPFC dopamine (DA) neurons firing rate between EtOH naïve, low alcohol drinking and high alcohol drinking mice (one-way ANOVA $F_{(2,2.149)}$=1.146, P=0.2585; Bonferroni multiple comparison's post-hoc test, p>0.05. n=10 cells/3 mice; n=12 cells/3 mice; n=11 cells/4 mice). Data represented as mean $\pm$ S.E.M.

Supplementary Figure 8

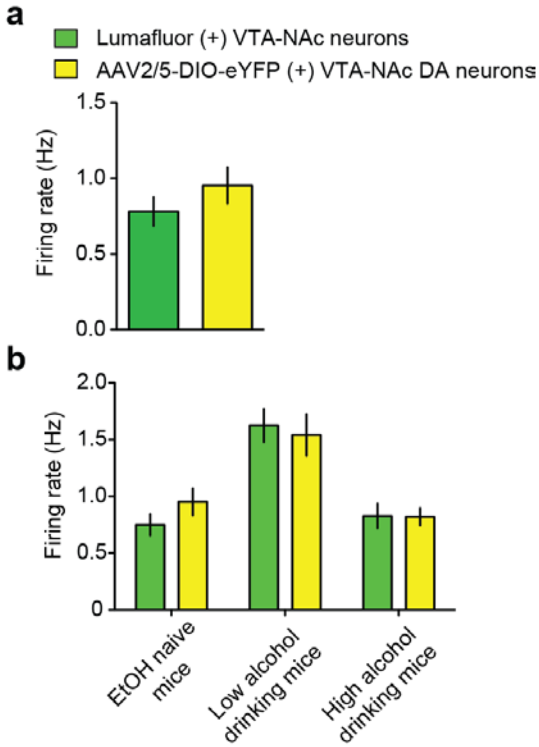

Supplementary Figure 8 No differences were observed between circuit-dissecting electrophysiological techniques. (**a**) Firing rate between Lumafluor labeled VTA-NAc (+) neurons and AAV2/5-DIO-eYFP labeled VTA-NAc dopamine (+) neurons in EtOH naïve mice (unpaired, two-tailed t-test: $t_{(37)}$=1.117, *P*=0.2712. *n*=24 Lumafluor(+) neurons, *n*=15 AAV2/5-DIO-eYFP(+) neurons). (**b**) Observations of VTA-NAc firing rate between groups infected with Lumafluor or with viral vector between alcohol drinking groups (two-way ANOVA: interaction effect $F_{(2, 107)}$=0.7116, *P*=0.4932; drinking group effect $F_{(2, 107)}$=20.60, *P*<0.0001; lumafluor vs DIO-eYFP effect $F_{(1, 107)}$=0.1329, *P*=0.7161; *n*=62 Lumafluor (+) neurons, *n*=46 AAV2/5-DIO-eYFP(+) neurons). Data represented as mean ± S.E.M.

Supplementary Figure 9

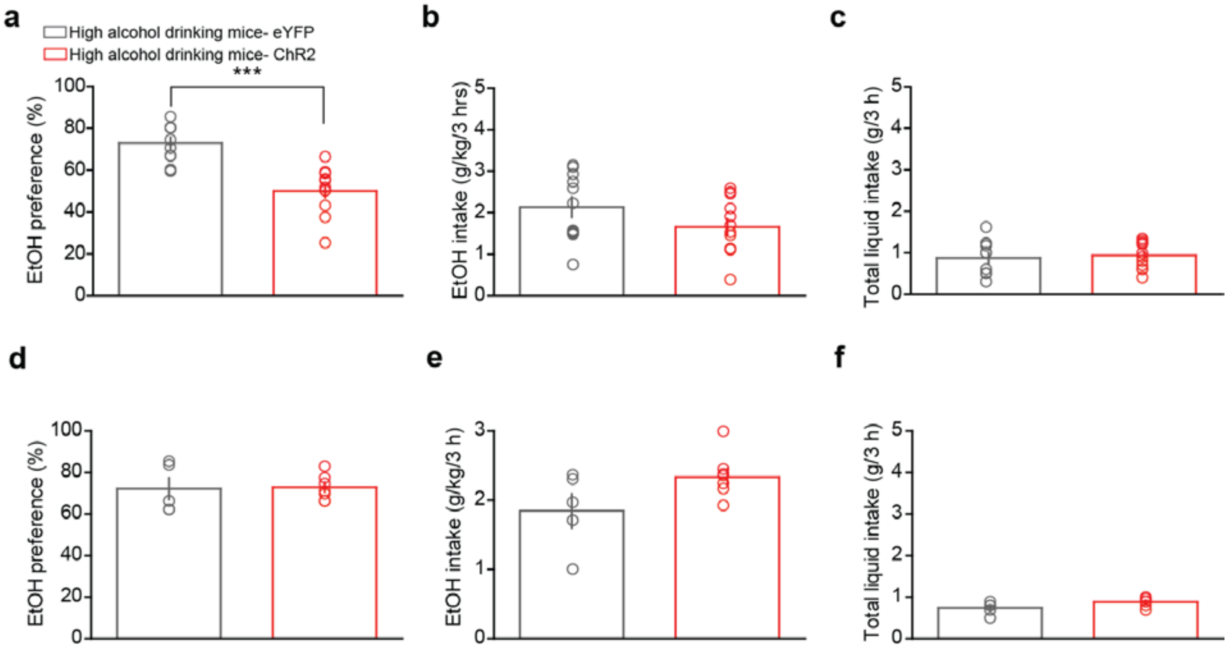

Supplementary Figure 9 3 h post stimulation alcohol drinking behaviors from projection-specific 15 min phasic stimulations. (**a**) EtOH preference of high alcohol drinking mice following VTA-NAc stimulation (unpaired, two-tailed *t*-test $t_{(21)}$=5.500, *P*<0.0001, *n*=11, *n*=12, ****P*<0.001). (**b**) EtOH intake of high alcohol drinking mice following VTA-NAc stimulation (unpaired, two-tailed t-test, $t_{(21)}$=1.5845, *P*=0.1373. *n*=11, *n*=12). (**c**) Total liquid intake of high alcohol drinking mice following VTA-NAc stimulation (unpaired, two-tailed t-test, $t_{(21)}$=0.5555, *P*=0.5844. *n*=11, *n*=12). (**d**) EtOH preference of high alcohol drinking mice following VTA-mPFC stimulation (unpaired, two-tailed t-test, $t_{(10)}$=0.1377, *P*=0.8932. *n*=5, *n*=7). (**e**) EtOH intake of high alcohol drinking mice following VTA-mPFC stimulation (unpaired, two-tailed t-test, $t_{(10)}$=1.930, *P*=0.0825. *n*=5, *n*=7). (**f**) Total liquid intake of high alcohol drinking mice following VTA-mPFC stimulation (unpaired, two-tailed t-test, $t_{(10)}$=1.934, *P*=0.0819. *n*=5, *n*=7). All data represented as mean ± S.E.M.

Supplementary Figure 10

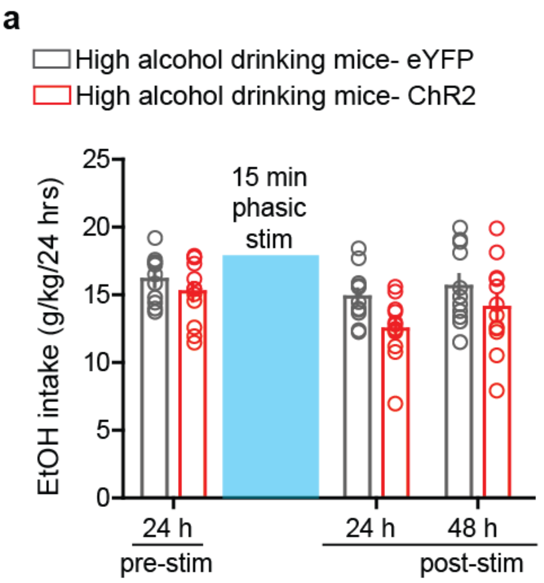

Supplementary Figure 10 EtOH intake behaviors of high alcohol drinking mice that had projection specific 15 min phasic stimulations. (**a**) EtOH intake of high alcohol drinking eYFP and ChR2 mice following 15 min of phasic VTA-NAc stimulation (two-way RM ANOVA: interaction effect $F_{(2,42)}=0.9185$, $P>0.05$; drinking group effect $F_{(1,42)}=3.807$, $P=0.0645$; time effect $F_{(2,42)}=7.036$, $P<0.01$; Bonferroni post-hoc test, eYFP vs ChR2 at 24 h $P>0.05$. $n=11$ mice; $n=12$ mice). (**b**) EtOH intake behaviors of eYFP and ChR2 high alcohol drinking mice following phasic stimulation of the VTA-mPFC pathway (two-way RM ANOVA: interaction effect $F_{(2,20)}=0.5885$, $P>0.05$; drinking group effect $F_{(1,20)}=1.285$, $P>0.05$; time effect $F_{(2,20)}=17.03$, $P<0.001$; Bonferroni post-hoc test, $P>0.05$. $n=5$ mice; $n=7$ mice). All data represented as mean ± S.E.M.

Supplementary Figure 11

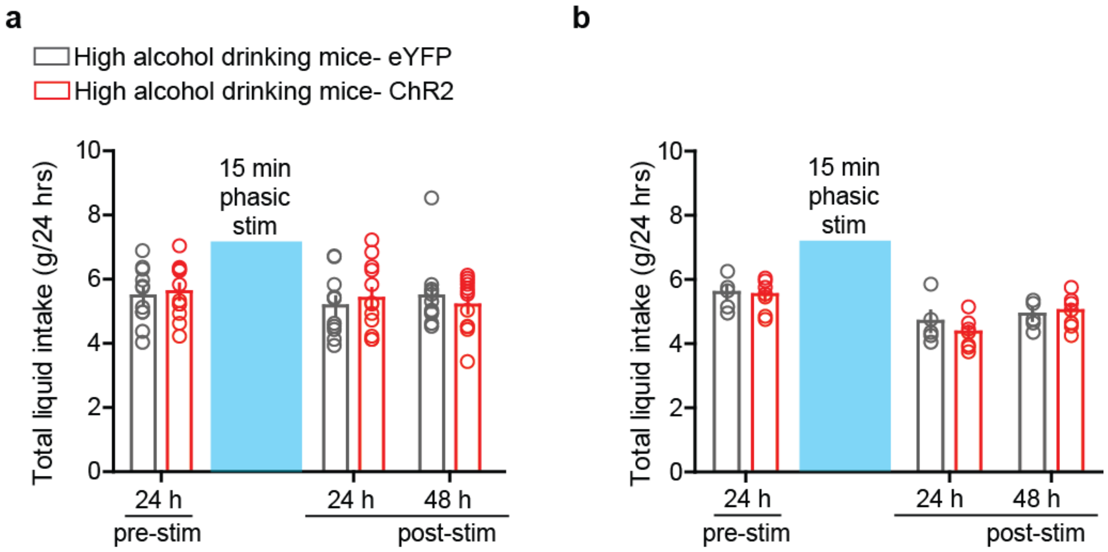

Supplementary Figure 11 Total liquid intake behaviors of high alcohol drinking mice that had projection specific 15 min phasic stimulations. (**a**) Total liquid intake of high alcohol drinking eYFP and ChR2 mice following 15 min of phasic VTA-NAc stimulation (two-way RM ANOVA: interaction effect $F_{(2,42)}=1.270$, $P>0.05$; drinking group effect $F_{(1,42)}=0.007528$, $P>0.05$; time effect $F_{(2,42)}=1.263$, $P>0.05$; Bonferroni post-hoc test, eYFP vs ChR2 at 24 h $P>0.05$. $n=11$ mice; $n=12$ mice). (**b**) Total liquid intake behaviors of eYFP and ChR2 high alcohol drinking mice following phasic stimulation of the VTA-mPFC pathway (two-way RM ANOVA: interaction effect $F_{(2,20)}=1.308$, $P>0.05$; drinking group effect $F_{(1,20)}=0.1403$, $P>0.05$; time effect $F_{(2,20)}=27.94$, $P<0.001$; Bonferroni post-hoc test, $P>0.05$. $n=5$ mice; $n=7$ mice). All data represented as mean $\pm$ S.E.M.

Supplementary Figure 12

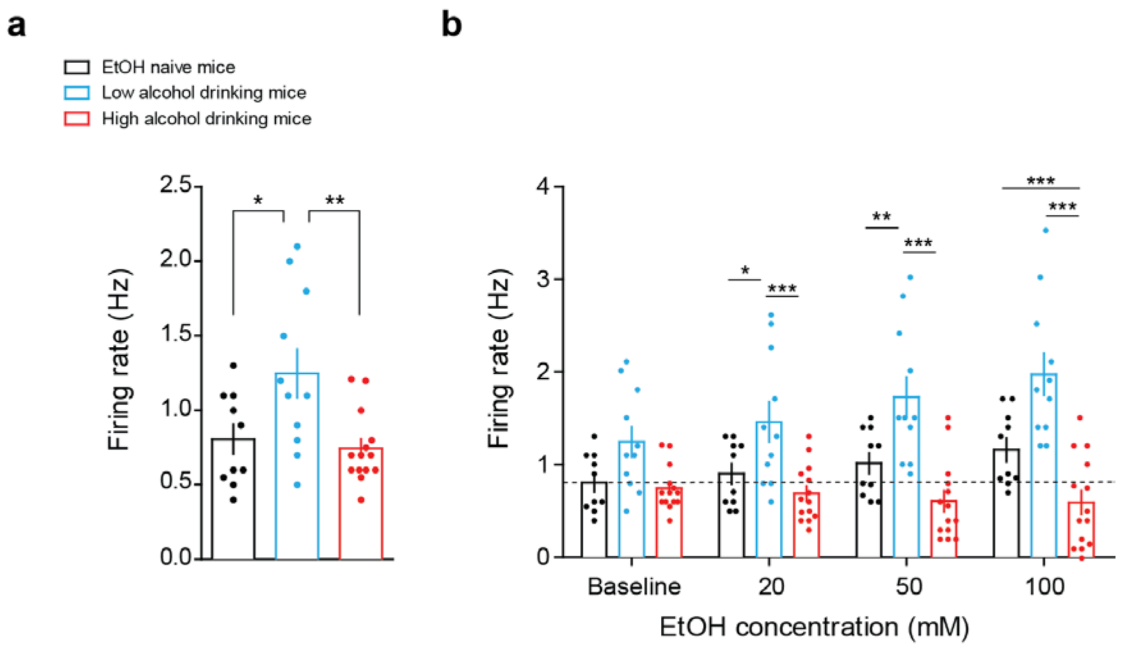

Supplementary Figure 12 Bath application of ethanol increases firing rate in ethanol naïve and low alcohol drinking mice. (**a**) Low alcohol drinking mice have significantly higher VTA dopamine (DA) neuron spontaneous firing rate during partial access recordings (one-way ANOVA $F_{(2, 1.733)}$=6.165, P<0.01, Bonferroni post hoc test **P*<0.05, ***P*<0.01. *n*=6 mice/10 cells; *n*=3 mice/11 cells; *n*=7 mice/14 cells). (**b**) Application of ethanol increases firing rate only in ethanol naïve and low alcohol drinking mice (two-way RM ANOVA: group effect $F_{(2, 96)}$=12.75, *P*<0.0001; EtOH concentration effect $F_{(3,96)}$=20.87, *P*<0.0001; interaction effect $F_{(6, 96)}$=16.07, *P*<0.0001. Bonferroni post hoc test, **P*<0.05, ***P*<0.01, ****P*<0.001. *n*=6 mice/10 cells; *n*=3 mice/11 cells; *n*=7 mice/14 cells)
